# Megabase-scale presence-absence variation with *Tripsacum* origin was under selection during maize domestication and adaptation

**DOI:** 10.1186/s13059-021-02448-2

**Published:** 2021-08-20

**Authors:** Yumin Huang, Wei Huang, Zhuang Meng, Guilherme Tomaz Braz, Yunfei Li, Kai Wang, Hai Wang, Jinsheng Lai, Jiming Jiang, Zhaobin Dong, Weiwei Jin

**Affiliations:** 1 State Key Laboratory of Plant Physiology and Biochemistry, National Maize Improvement Center, Key Laboratory of Crop Heterosis and Utilization (MOE), Joint International Research Laboratory of Crop Molecular Breeding (MOE), China Agricultural University, Beijing, 100193 China; 2 Key Laboratory of Genetics, Breeding and Multiple Utilization of Corps (MOE), Fujian Agriculture and Forestry University, Fuzhou, 350002 Fujian China; 3 Department of Plant Biology, Department of Horticulture, Michigan State University, East Lansing, MI 48824 USA

**Keywords:** Maize, *Tripsacum*, Presence-absence variation, NOR, Domestication

## Abstract

**Background:**

Structural variants (SVs) significantly drive genome diversity and environmental adaptation for diverse species. Unlike the prevalent small SVs (< kilobase-scale) in higher eukaryotes, large-size SVs rarely exist in the genome, but they function as one of the key evolutionary forces for speciation and adaptation.

**Results:**

In this study, we discover and characterize several megabase-scale presence-absence variations (PAVs) in the maize genome. Surprisingly, we identify a 3.2 Mb PAV fragment that shows high integrity and is present as complete presence or absence in the natural diversity panel. This PAV is embedded within the nucleolus organizer region (NOR), where the suppressed recombination is found to maintain the PAV against the evolutionary variation. Interestingly, by analyzing the sequence of this PAV, we not only reveal the domestication trace from teosinte to modern maize, but also the footprints of its origin from *Tripsacum*, shedding light on a previously unknown contribution from *Tripsacum* to the speciation of *Zea* species. The functional consequence of the *Tripsacum* segment migration is also investigated, and environmental fitness conferred by the PAV may explain the whole segment as a selection target during maize domestication and improvement.

**Conclusions:**

These findings provide a novel perspective that *Tripsacum* contributes to *Zea* speciation, and also instantiate a strategy for evolutionary and functional analysis of the “fossil” structure variations during genome evolution and speciation.

**Supplementary Information:**

The online version contains supplementary material available at 10.1186/s13059-021-02448-2.

## Introduction

A striking range of natural genetic diversity has been revealed at the level of SNPs, indel polymorphisms (IDPs), and structural variation [1–4]. Structural variation involves an order of magnitude higher base pairs relative to SNP, which is the largest source of genetic variation [4, 5]. Structural variation mainly includes presence-absence variants (PAV), copy number variants (CNV), inversions, translocations, and more complex rearrangement [6, 7]. One extreme form of CNV is the presence-absence variation (PAV), in which a particular sequence is present in some individuals while absent in others. The advance of long- and short-read sequencing technologies has significantly boosted the identification of PAVs in numerous species [8–10]. However, the prevalence of PAVs in the genome-wide context varies among species as well as individual samples. For example, PAVs have been prevalently identified in bacterial genomes since firstly found in the prokaryotes decades ago [11, 12], and the intraspecific PAVs in bacteria could count for over half of the genome [13]. In contrast, in eukaryotes particularly in higher animals including humans, PAVs were found often with a much smaller size scale, and they predominantly consist of intergenic sequences [14, 15]. Nevertheless, a growing number of eukaryotes, such as fungi, algae, and plant species, have been reported with large-size PAVs containing functional genes [9, 10, 16–19].

Compared to small PAVs, large-size PAVs bring instability of the genome, possibly resulting in their failure in the transmission to the subsequent generations [20]. Coding genes and/or regulatory elements within large PAVs may lead to deleterious or even lethal lesions for biological function or genome integrity, consequently showing vulnerability to evolution purification in the diversity population [16]. More importantly, even if large variations could confer elevated fitness and thus escape from sequence divergence and erosion, it is almost inevitable that they will gradually be rearranged into smaller-sized variations, as a result of intraspecific homologous recombination [21].

Multiple mechanisms have been proposed for the PAV formation in the genome, such as the copy/cut-paste mechanism by transposable elements and improperly paired meiotic recombination [22, 23]. These processes, however, antagonized by the genome repair mechanism, may not be effective to raise large-size PAVs. Instead, interspecific gene transfer and genome fractionation following duplication have been reported as the important ways to generate large PAVs [24, 25]. For example, bacteria can either incorporate exogenous DNA into their own genomes, or exchange genetic elements between individuals, leading to prevalent large-size PAVs [26]. In plant species, interspecific hybridization is ubiquitous during speciation [27, 28]. The interspecific hybridization generates large PAVs, likely due to the homologous exchange between the progenitor genomes [29–32]. It has been shown that large-size PAVs in several polyploidy crops were raised in the process of polyploidization and the subsequent genome fractionation [33]. Therefore, large PAVs could be an excellent system to infer novel insights for speciation and evolution. In fact, SVs play critical roles in understanding the genetic contributions of plant domestication history and phenotypic plasticity [34]. However, due to the current lack of evaluation of large-size PAVs in plants and their own rarity, it is still ambiguous if interspecific transfer or genome fractionation directly links to the large-size PAVs.

Maize (*Zea mays* ssp. *mays*) is an ancient tetraploid [35, 36]. The polyploidization, chromosome rearrangement, and the following genome fractionation attributed to the *Zea* speciation are believed to generate prevalent SVs including large PAVs [37, 38]. The maize genome is highly malleable, which can also be described by a surprisingly large amount of SV [39–41]. Indeed, nearly 4000 instances of CNV/PAV were observed in a diversity panel of maize and the wild ancestor teosinte lines [42], although their dynamics underlying speciation and the later domestication are still largely unknown. Modern maize was domesticated from teosinte (*Zea mays* ssp. *parviglumis*) and about 2 to 4% of all maize genes experienced artificial selection [43]. Along with that, significant introgression has also been reported with genomic fragments flowing from another teosinte sister species *Zea mays ssp. mexicana* [44, 45]. The potential gene flow from other *Zea* species that are interfertile with maize, such as *Zea luxurians*, possibly has also contributed to the maize genome evolution and domestication [46]. Outside the *Zea* genus, the closest relative is the genus *Tripsacum* [47], which had been notoriously proposed as the domestication origin for maize until the role of teosinte was confirmed [48]. Rare cases of natural and artificial *Zea-Tripsacum* hybrids have been identified, but the offspring are invariably sterile [48, 49]. Nevertheless, there has been little evidence to rule out the possibility that *Tripsacum* was interfertile with the *Zea* ancestor in ancient times. Investigation on the potential PAVs directly resulted from the interspecific introgression could be a promising strategy to sketch the process through *Zea* speciation to domestication.

An exceptional PAV at megabase scale has been revealed between two heterotic inbred lines B73 and Mo17 [50, 51]. The recent revolutionary sequencing strategy using single-molecule technologies has improved the quality of the de novo genomes of B73 and Mo17, providing extensive information on the genome-wide variations between these two inbreds [3, 52]. However, the insights on the genomic and evolution signature, and the functional consequence of the largest PAVs that contains hundreds of genes in these regions, are still intriguing questions to be investigated. Here, we conducted comparative genomics analysis of B73 and Mo17 and documented a 3.2 Mb PAV within the nucleolus organizer region (NOR) on chromosome 6. The entire sequence of this PAV is largely intact in a diversity panel ranging from teosinte to maize, likely due to the suppressed recombination by NOR to counteract the evolution sequence erosion. A series evidence indicated that this PAV originated from *Tripsacum*, providing direct support for the contribution of *Tripsacum* to the *Zea* speciation. Lastly, we found this *Tripsacum*-originated PAV may be consistent with selection during maize domestication and improvement, by having potential function on environmental adaptation.

## Results

### Fine mapping of megabase-scale PAVs between maize inbreds B73 and Mo17

To capture large-size PAVs in the genome context of maize inbred lines, we compared the recent genome assembly of B73 and Mo17, which have been recently improved by single-molecule sequencing technique [3, 52]. We aimed to isolate the large PAVs with sizes over 500 kb that are present in one inbred while absent in the other. Here we used a sliding-window method that was previously reported which divided one reference genome into 500-bp windows with a step size of 100 bp and aligned to another genome; the windows that could not be aligned to the other genome were merged within 100 kb of the physical coordinates and then defined as a PAV cluster [3]. Three regions (designated as RegionA, RegionB, and RegionC hereinafter, with a size of 2.9 Mb, 628 kb, and 547 kb, respectively) were found present only in B73, while two regions (designated as RegionD and RegionE with a size of 2.5 Mb and 753 kb, respectively) were found only in Mo17 (Additional file 2: Table S1); these results are consistent with the previous study [3].

Using the recent publicly available Hi-C data for B73 chromatin architecture and the newly improved B73 AGPv4 genome as a reference [52, 53], we were able to validate the physical location and orientation of these three large PAVs in B73. Consistent with the genome-wide profiling that chromatin interaction matrix substantially matched with genome assembly, the two smaller PAVs, RegionB and RegionC, were verified based on the consistency between the Hi-C data and genome reference (Additional file 1: Figure S1a, b). However, an abnormal interaction was observed between 22 M to 25 M on chromosome 6 based on AGPv4 genome, which maps to the exact location of the largest PAV RegionA (Additional file 1: Figure S1c), suggesting a putative assembly error in this region. Therefore, we revisited this scaffold and indeed found a Bionano map error. Eventually, an updated version of assembly for this region was rectified and designated as AGPv4.1, based on which the RegionA location was adjusted to the physical interval approximately between 13 M and 16 M on Chromosome 6 (Additional file 1: Figure S1d, e). As a consequence of the improved accuracy of AGPv4.1, the length of RegionA was determined to be 3.2 Mb, which was slightly expanded than the previous reports [3, 50]. In total 70 protein-coding genes were annotated in this region (Additional file 2: Table S1).

We remapped Mo17 resequencing data [54] against the B73 genome reference and vice versa and observed nearly all the annotated genes (12/13, 6/8, and 13/20) in RegionB, RegionC, and RegionE had the homologous alleles in the genome of the absence counterpart (CDS coverage > 80%), suggesting these PAVs may be caused by translocation rather than deletion. In contrast, for both of the megabase PAVs, 65 out of 70 RegionA genes and 9 out of 10 RegionD genes were found completely missing in the Mo17 and B73 genome (CDS coverage < 50%) respectively, suggesting that gene sets contained in RegionA and RegionD are true presence-absence alleles.

**Fig. 1:**
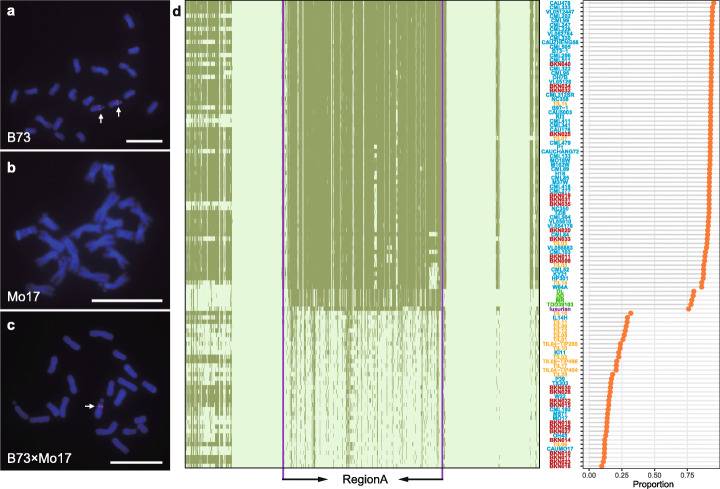
Characterization of a megabase-scale PAV, RegionA, in diverse maize inbreds and their wild relatives. **a–c** Fluorescence in situ hybridization (FISH) of RegionA-specific oligo probes on root tip metaphase chromosomes of B73, Mo17, and their F1 hybrid. RegionA-specific signals (red) were observed on a pair of homologous chromosomes of B73 metaphase cells (**a**), while absent on Mo17 metaphase chromosomes (**b**). Only one foci signal can be detected on mitotic metaphase spreads of B73 × Mo17 F1 hybrid (**c**). Scale bars = 10 μm. **d** Left: PAV map of RegionA and its flanking regions (2 Mb on each side) in diverse maize inbreds and their wild relatives. Each column represents a bin (dark green for presence bin, light green for absence bin), and each row represents a line (text color of green for *Tripsacum*; purple for *Zea luxurians*; yellow for teosintes; red for maize landraces; blue for maize inbreds). Right: The proportion of presence bins in RegionA for each line

We designed a FISH probe using single-copy oligos within the RegionA region. The oligo-FISH signals were detected in the expected region on Chromosome 6 in B73 (Fig. 1a); in contrast, no signals were detected in Mo17 (Fig. 1b), substantiating the absence of RegionA in Mo17. Consistently, the B73/Mo17 F1 hybrids showed FISH signal only on the B73 chromosomes while missed on the Mo17 allelic location (Fig. 1c).

### PAVs RegionA and RegionD present in the diversity population as integrated elements

We next investigated the prevalence of these large PAVs in the maize natural variation population. A set of genome resequencing data was used for analysis, including 17 teosinte lines (2 *Zea* ssp. *mexicana* and 15 *Zea* ssp. *parviglumis*), 23 landraces, 60 maize inbreds [55], and 1 *Zea luxurians* line [56]. In addition, as sister genus related to *Zea*, 4 *Tripsacum* [57] and 9 sorghum lines [58] were also included (Additional file 2: Table S2). Through a 10-kb sliding window analysis with a step size of 1 kb, all five large PAVs between B73 and Mo17 were characterized in the above-mentioned accessions (Fig. 1d, Additional file 1: Figure S2 and 3). B73 PAVs RegionB, RegionC, and Mo17 PAVs RegionE showed a continuous distribution of presence-bin proportion among the diversity lines (Additional file 1: Figure S2a,b and Additional file 1: Figure S3b), suggesting extensive divergence in these PAV regions.

In contrast, the two megabase-scale PAVs, RegionA of B73 and RegionD of Mo17, showed a surprisingly polarized pattern of genotype distribution: contrasting groups of lines with presence-bin proportion of either less than 30% or larger than 75%; besides that none of the lines were found to contain an intermediate presence-bin proportion (Fig. 1d and Additional file 1: Figure S3a). In addition to the above genome resequencing data, the stable feature of both RegionA and RegionD was further confirmed by the syntenic comparison between multiple inbreds with *de novo* genome assembly including 26 high-quality NAM founders genome using PacBio long reads, a mate-pair strategy [59–61]. B73 RegionA showed well collinearity with the genome of PH207, B104, and 19 out of 24 other NAM founders besides B73 (Additional file 1: Figure S4), but no collinearity with Mo17, W22, and remaining NAM founders, consistent with the presence-bin proportion pattern shown in Fig. 1d. We further investigated the coding sequence coverage of RegionA genes by mapping HapMap2 resequencing reads to B73 reference genome, and the results showed that different from RegionB and RegionC, the majority of RegionA genes are nearly completely present or absent in diversity lines (Additional file 1: Figure S5a). In accordance with that, FISH experiment using RegionA-specific oligo probes showed signals in the RegionA-present inbreds while none detected in the absent lines (Additional file 1: Figure S5b-e). These results suggested an unexpected scenario that the majority of RegionA and RegionD appear to be present or absent as an integrated element among the diversity panel, only experiencing extremely low levels of internal sequence divergence within the region.

### RegionA infers an origination from *Tripsacum*

Given the integrated feature of RegionA and RegionD, we grouped the diversity accessions based on the presence or absence of the PAVs. RegionD appeared to be a relatively rare allele, present only in 10% of the accessions tested in this study, including two inbreds (Mo17 and Ms71) and several lines of landrace and teosinte. In contrast, the frequency of RegionA is much higher, as it is present in 66% of all the accessions tested, including most inbreds, several landraces, and teosinte lines (Fig. 1d). We also identified the RegionA genotypes in the diversity panel of maize, and the results showed that no RegionA heterozygotes were found in 100 HapMap2 lines, but 26 of 99 Meso- and South America lines were defined as RegionA heterozygotes (Additional file 1: Figure S6). Interestingly, 4 of 4 (100%) *Tripsacum* and the one *Zea luxurians* contained major portions (76% ~ 80%) of RegionA (Fig. 1d), suggesting the emergence of this element predated the divergence of *Zea* and *Tripsacum* from their common ancestor. Nevertheless, RegionA was not observed in the 9 sorghum lines (Additional file 1: Figure S2c), and no collinearity of RegionA was found in more distantly related species, including *Setaria* and rice (Additional file 1: Figure S4). Though we cannot rule out the possibility that RegionA may also be present in a more distant genus but undetectable due to the lack of a corresponding genome, our result to date indicates RegionA appears as a specific element for the *Tripsacinae* subtribe.

The *Tripsacinae* specific sequence of RegionA inspired us to trace the origin of this largest PAV. The long terminal repeat retrotransposon (LTR-RT) is the most abundant repeat type in the maize genome, and the divergences of LTR retrotransposons have been widely used to estimate the date of insertion for landmarking the evolution of flanking region [62]. Approximately 68% of transposable elements (TE) were annotated as LTR-RTs in RegionA, a proportion similar to the genome-wide level (Additional file 1: Figure S7a,b). Nevertheless, the RegionA was found to consist of more unknown superfamily retrotransposons (RLX) compared to the average genome-wide level (Additional file 1: Figure S7c). Besides, almost 70% of the LTR-RTs were annotated as non-maize-family (Additional file 1: Figure S7d). By calculating the LTR insertion time in RegionA, we estimated that the oldest insertion event occurred approximately 1.74 Mya (Additional file 1: Figure S7e). This is earlier than the divergence of *Zea* from *Tripsacum* genus which was estimated no earlier than 1.2 million years ago [63]. This result suggested that RegionA, or at least parts of this element, can be traced back to the common ancestors of *Tripsacum* genus and *Zea* genus.

**Fig. 2:**
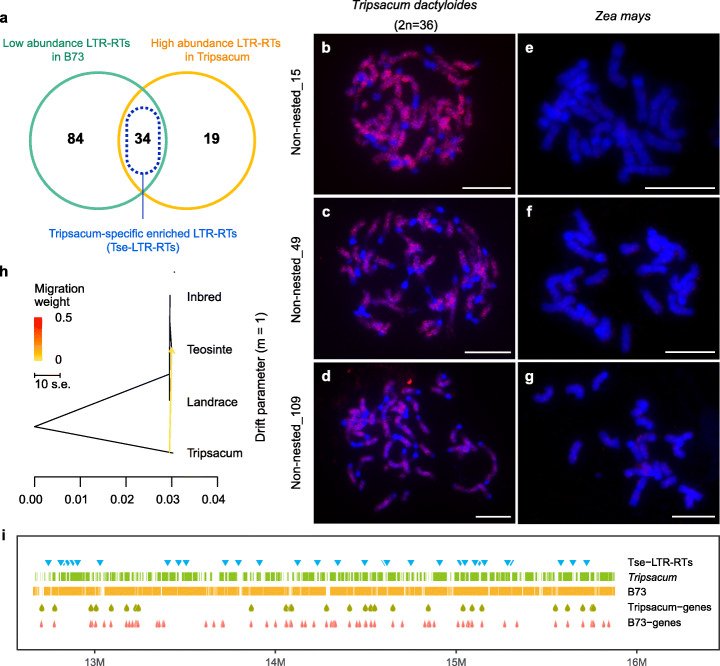
RegionA is enriched with *Tripsacum*-specific LTR retrotransposons. **a** Venn diagram of different types of LTR-RTs in RegionA. The intersection (blue dotted frame) of low abundance LTR-RTs in B73 and high abundance LTR-RT in *Tripsacum* represent *Tripsacum*-specific enriched LTR-RTs (Tse-LTR-RTs). **b–g** FISH analysis of the distribution of three *Tripsacum*-specific enriched LTR retrotransposons (Tse-LTR-RTs) cloned from RegionA, all three Tse-LTR-RTs showed genome-wide distribution in *Tripsacum dactyloides* (2n = 36) (**b–d**), while they had no obvious signal in *Zea mays* (**e–g**). Scale bars = 10 μm. **h** Gene flow detected between *Tripsacum* and *Zea* in RegionA. One migration event was set for analysis (m = 1), direction of gene flow was indicated by an arrow. **i** Schematic Structure of LTRs and genes in RegionA. Rows from top to bottom indicate location of Tse-LTR-RTs; read coverage of *Tripsacum* and B73; location of *Tripsacum* consistent genes and B73 annotation genes, respectively

As types of LTR-RTs show various abundance in the genome, we examined the genome-wide abundance of the corresponding LTR-RTs types in RegionA (see “Methods”) and found a major proportion of RegionA LTR-RTs represents the types with low genome-wide abundance (Additional file 1: Figure S7f). This result suggested that despite the ancient nature of this region, the containing LTR-RTs were predominantly trapped in the local region rather than expanding to the global genome. All the LTR retrotransposons in RegionA were then categorized by the abundance in the context of the *Zea* and *Tripsacum* genome background, respectively (Additional file 2: Table S3). The low abundance LTR retrotransposons relative to the B73 surprisingly shared an intensive identity with the highly abundant LTR retrotransposons in the *Tripsacum* genome (designated as *Tripsacum*-specific enriched LTR retrotransposons, or Tse-LTR-RTs hereafter) (Fig. 2a, i). To visualize the genomic distribution of the Tse-LTR-RTs between maize and *Tripsacum*, we developed probes from three selected Tse-LTR-RTs for FISH analysis. As expected, the Tse-LTR-RTs probes showed strong hybridization signals throughout the *Tripsacum* chromosomes, whereas no unambiguous signals were detected on maize chromosomes (Fig. 2b–g, Additional file 1: Figure S7g-i). Interestingly, a previous study has isolated several *Tripsacum*-specific retroelements that showed specific FISH hybridization in *Tripsacum* lineage but not in the *Zea* species [64]. We validated these *Tripsacum*-specific retroelements by BLAST search against the B73 genome and filtering with stringent parameters, and found all perfectly matched loci were located exclusively in RegionA (Additional file 2: Table S4), substantiating the enrichment of *Tripsacum* LTR-RTs in RegionA. Additionally, evidence from homologous coverage and genes also highlighted the major contribution from *Tripsacum* to this region (Fig. 2i).

Among the LTR retrotransposons in RegionA, the Tse-LTR-RTs were estimated to be older than the remaining retrotransposon counterparts, suggesting the *Tripascum*-originated components may serve as the initial foundation for the later divergence of this region (Additional file 1: Figure S7j). This intrigued us to investigate whether RegionA was originally raised by a transfer from ancient *Tripsacum*, as interspecific transfer has been instanced to generate PAVs in plant genome [65]. Indeed, a treemix analysis identified signals for RegionA introgression from *Tripsacum* to *Zea* (Fig. 2h, Additional file 1: Figure S8a,b). This introgression from *Tripsacum* appeared unique only within RegionA, since the same analysis using the whole chromosome 6 as inputs only showed intraspecies gene flow among *Zea* genus whereas the introgression from *Tripsacum* was not detectable (Additional file 1: Figure S8c-e). The analysis for absolute sequence divergence (*d*_*xy*_) has been generally used as the benchmark for distinguishing recent gene flow from shared ancestral variation [66, 67]. We measured the *d*_*xy*_ of RegionA between *Tripsacum* and *Zea*. Compared with the rest of the genome, RegionA showed a dramatically reduced *d*_*xy*_ that visualized as a pattern of trough on the entire chromosome (Additional file 1: Figure S9a), and the *d*_*xy*_ value of 60.5 ~ 72.2% RegionA windows was below the cutoff of whole-genome bottom 5% (Additional file 1: Figure S9b). This result shows a distinct signal of reduced divergence between *Tripsacum* and *Zea* at RegionA, which is likely due to the recent coalescence at this region led by introgression. We attributed the exclusive detection for *Tripsacum* origin to the characteristic integrity of RegionA, as the ancient flow in RegionA is still well preserved while the rest of the genome has been shuffled. These results proposed the segment transfer between the two sister species, arguing a possible direct contribution from *Tripsacum* in the speciation of *Zea* family.

### Suppressed recombination by 45S rDNA contributed to maintaining the integrity of RegionA

We wondered if RegionA and RegionD are located in recombination-suppressed regions, which may result in the intriguing integrity of these sequences in the diversity panel. Indeed, RegionD was found located in the pericentromeric region of Chromosome 6, characterized with a low rate of recombination [68].

**Fig. 3:**
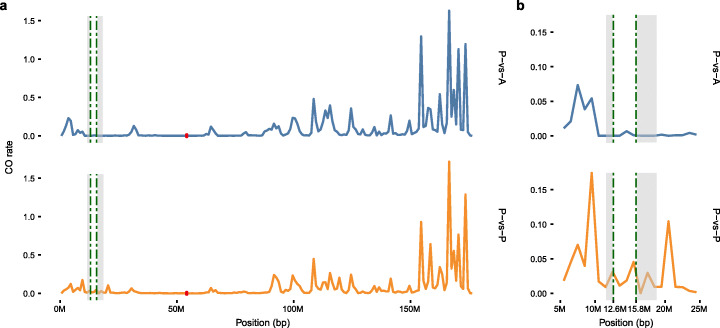
Comparison of crossover landscape of chromosome 6 in the presence versus absence (P-vs-A) population (*n* = 1198) and presence versus presence (P-vs-P) population (*n* = 3786). All lines were isolated from the nested association mapping population. Green dash lines indicate the RegionA, gray blocks indicate the gaps near RegionA, red dots indicate the centromere region. **a** Whole chromosome 6. **b** A zoomed-in view of the region from 5 to 25 Mb

**Fig. 4:**
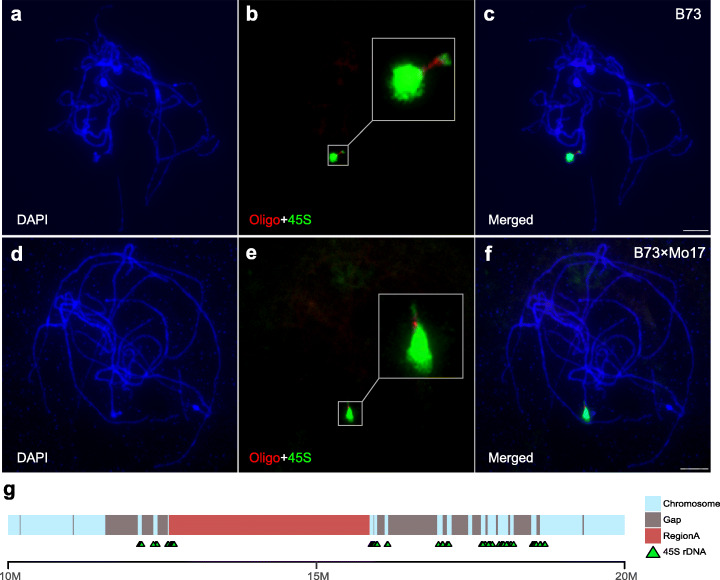
RegionA is embedded in the 45S rDNA region. **a–f** FISH analysis of the relative distribution of 45S rDNA (green, marked as 45S) and RegionA (red, marked as Oligo) on the chromosome 6 of B73 (**a–c**) and B73 × Mo17 (**d–f**) meiotic pachytene chromosomes. Scale bars = 10 μm. **g** Schematic Structure of RegionA and flanking regions (from 10 to 20 Mb based on maize B73 assembly v4.1). The red block represents the RegionA, green triangular indicate the location of 45S rDNA, and light blue bars represent the reminder region while the dark gray represent unfilled gaps in the current genome assembly

RegionA, in contrast, is located on the distal end of Chromosome 6 short arm, far from the pericentromere; therefore, this region was generally supposed to have a high rate of recombination. However, the short arm of Chromosome 6 is known to carry the 45S rDNA and a chromosomal “satellite”, and it is also associated with a low level of crossovers (Fig. 3a) [68–70]. The low recombination rate in this area was believed to be caused by the nucleolus organizer region (NOR) located in this region [70, 71]. Due to the highly repetitive nature of the NOR, this region is poorly assembled. Even in our updated AGPv4.1, this region still contains numerous gaps (Additional file 1: Figure S1d). To determine the accurate location of RegionA relative to the NOR, FISH assay was performed in B73 and B73 × Mo17 hybrid meiotic pachytene using an oligonucleotide-based probe specific to RegionA (see “Methods”) together with a 45S rDNA probe. The FISH results indicated that RegionA was embedded within the 45S rDNA arrays and was mapped toward the proximal end of NOR (Fig. 4a–f). Mapping 45S rDNA sequence back to the AGPv4.1 genome also revealed the presence of 45S rDNA sequences around the RegionA (Fig. 4g). Note that the schematic amount of 45S rDNA was possibly underestimated due to the unfilled gaps.

It has been widely acknowledged that structural variations, including PAVs, also strongly affect the frequency and distribution of recombination events in animals and plants [72, 73]. Despite the low recombination rate around the NOR, we noticed that the recombination rate was even more suppressed in the NAM RILs from RegionA-absence founder pairs, in contrast to that from all RegionA-presence founders (Fig. 3b). Therefore, the suppressed recombination, caused by a heterochromatic chromosomal environment as well as the structural variation itself, may play a major role, to maintain the intact status for megabase-scale RegionA and RegionD during million years of evolution.

### RegionA was under selection during maize domestication and improvement

**Fig. 5:**
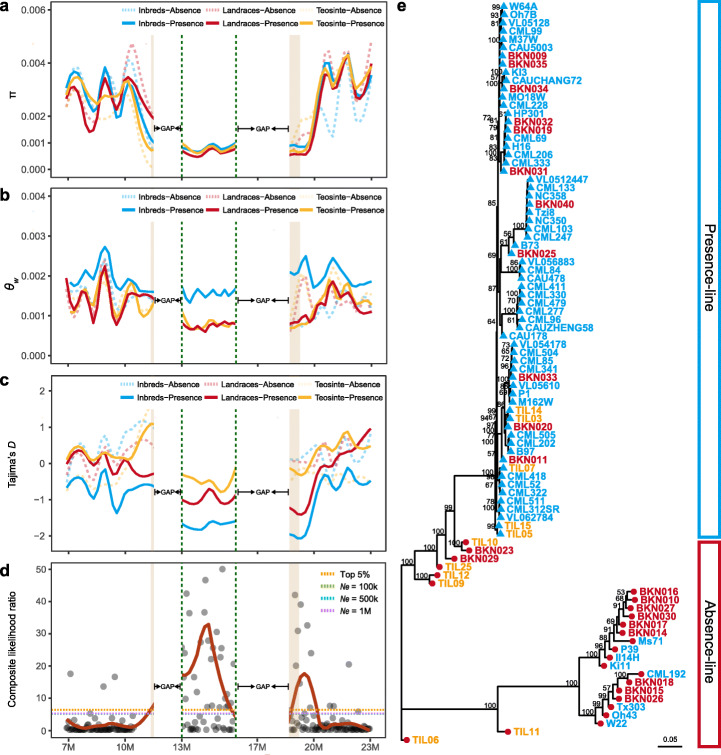
Genetic diversity of RegionA in the context of domestication. **a–d** RegionA harbors low genetic diversity and positive selection signals inside. Four indicators of sequence diversity and selection signals, nucleotide diversity (π), Watterson’s estimator (*θ*_*W*_), Tajima’s *D*, and composite likelihood ratio (CLR) of modern inbreds were shown in **a**, **b**, **c**, and **d**, respectively. The 100 kb upstream and 500 kb downstream flanking regions were indicated by brown blocks. The two green vertical lines indicate RegionA, and indicators were calculated only based on presence line; for **d**, dashed horizontal lines indicate the cutoff of composite likelihood ratio. **e** Neighbor-joining tree of the HapMap lines in flanking regions of NOR (upstream 100 kb and downstream 500 kb). The bootstrap values on the tree are based on 1000 replicates and values more than 50 are displayed. Taxa in the tree are represented by different text colors: teosintes (yellow), landraces (red), and improved lines (blue). The tree tip points labeled by blue triangles indicate the RegionA-presence lines, while red circles indicate the absence lines

To obtain genetic insights into RegionA and domestication, we surveyed polymorphism and divergence at this region with different subgroups. Average overall π (nucleotide diversity) and *θ*_*W*_ (population mutation rate) for teosinte, landrace, and inbred in RegionA are 0.00079, 0.00066, 0.00083 and 0.00083, 0.00079, 0.00160, respectively. They are all significantly lower than the level of whole chromosome 6 (Fig. 5a,b). Within RegionA, pre- and post-domestication lines showed little evident difference of nucleotide diversity (Fig. 5a), but a relatively lower level of population mutation rate was found in teosinte and landrace compared to inbred (Fig. 5b).

Since RegionA has been extremely conserved due to the suppressed recombination brought by NOR, we wonder whether this effect spreads to the peripheral area of NOR. Here we made the pairwise comparison of nucleotide diversity between RegionA and upstream or downstream flanking regions of NOR with different scales. We found that the π value showed no difference between RegionA and upstream 100 kb and downstream 500 kb (Additional file 1: Figure S10a,b), while inside these two regions there was almost no difference between RegionA-presence lines and absence lines (Additional file 1: Figure S10c,d).

To further investigate the phylogenetic relationships among diversity panels in RegionA, we extracted the SNPs of the flanking region mentioned above for phylogenetic tree construction. We can see the tree showed that most teosinte lines were basal to the maize lines, while diversity lines were completely separated into two major clusters according to the presence or absence of RegionA (Fig. 5e), which means the NOR flanking region and RegionA as a whole, showed a distinct evolutionary relationship from the entire genome.

RegionA is present in 5 of 17 (29%) teosinte, indicating it is a standing genetic variant in teosinte. But this frequency rose to 11/23 (48%) and 50/60 (83%) in landrace and maize inbred line respectively, exhibiting a progressively increasing enrichment along with the maize domestication and improvement (Fig. 1f). To examine whether RegionA has been under selection during domestication or modern breeding, a series analysis was conducted. For Tajima’s *D*, there is an excess of negative values in RegionA for maize landraces and inbreds (− 0.9695, − 1.7032, respectively, Fig. 5c), which suggests an overall excess of low-frequency mutations. Though the negative value was interpreted as evidence for positive selection, the demographic factors, background selection, or a combination of these factors can also affect the results. Here we scanned the signatures of selective sweeps for RegionA-presence maize landraces and inbreds, using a site frequency spectra (SFS)-based method by SweeD [74]; the composite likelihood ratio (CLR) was calculated with grid sizes of 50 kb across each chromosome. We then determined the significance threshold to reject the null model of neutrality based on the demography of maize domestication by coalescent simulations [75]. Finally, we found the significant signature of selection inside the RegionA for landrace and inbred (Fig. 5d, Additional file 1: Figure S10e-g), which rejected the null model of neutrality. Taken together, all the above evidence provided the support that RegionA was targeted by positive selection.

### RegionA genes show presence-absence expression patterns

As a consequence of the PAVs between the inbred lines, the reciprocal hybrids have only haploid allele from the presence parent, in contrast to the diploid genome background with both parental alleles, leading to a scenario of segmental dosage. We investigated the transcription of genes located within RegionA using publicly available transcriptome data generated from the seedling shoot (diploid cells) and endosperm (triploid cells) tissue of B73, Mo17, and their reciprocal hybrids (Additional file 2: Table S4) [76–80]. RegionA genes tended to show approximately a 50% expression level in BM or MB hybrid relative to B73 in the diploid tissue types (Additional file 1: Figure S11a-c). In the endosperm tissue, the majority of the RegionA genes showed a fold change of log_2_(2/3) when B73 as the maternal parent, and log_2_(1/3) when B73 as the paternal parent. The change of these RegionA genes was linearly consistent with the dosage ratio asymmetrically contributed by B73 to this triploid tissue (Additional file 1: Figure S11d-f). We also examined the epigenetic modification on these PAV genes, including the positively transcriptional marker H3K4me3, H3K36me3, and H3K9ac [77, 79]. We found that the reduction of modifications was closely correlated with the dosage of transcription activity (Additional file 1: Figure S12).

It is worth noting that there are a number of non-additive B73-specific PAV genes in RegionA (19/43), which showed extremely low or high dosage effects in at least one tissue. These RegionA non-additive genes generally showed a high tissue specificity (Additional file 1: Figure S13), and their proportion relative to the additive genes showed mild but significant differences in some tissues compared with that of the genome-wide level (Additional file 2: Table S5). Given the non-additive effect has been acknowledged as a result of the genetic interaction and dosage compensation [81–83], the annotation of these genes implied the potential roles of these RegionA genes in diverse biological processes, such as development, stress response, and chromatin interaction. (Additional file 2: Table S5).

### RegionA genes are associated with environmental fitness

Since the megabase-scale PAV RegionA appeared under strong selection, we explored the underlying function for the local genes, which may serve the biological foundation of the selection. However, compared to a genome-wide level, RegionA significantly enriched more genes that lack Gene Ontology (GO) annotation (Additional file 2: Table S7). In addition, approximately 40% of the genes in RegionA had no ortholog found in the species outside the *Tripsacinae* subtribe (Additional file 2: Table S7), obscuring our understanding of the functions of these genes.

We designed a different approach to explore the possible function of RegionA genes by examining the transcriptome changes affected by RegionA. From B73-Mo17 Near-Isogenic Lines [84], we selected two NILs, and referred to as B^M^ for a line with Mo17 RegionA loci introgressed into B73-like background, and as M^B^ for a line vice versa (Additional file 1: Figure S14a,b). Transcriptome analysis was conducted for both NILs, as well as the B73 and Mo17 parental inbreds as controls. The presence or absence of RegionA resulted in only a subtle change in the genome-wide context, as the NILs showed a highly similar transcriptome as their respective parental inbred (Additional file 1: Figure S14c). Nevertheless, a conspicuous proportion of the transcriptome, 2592 genes and 2151 genes, respectively, was identified as differentially expressed genes (DEGs) in B^M^ and M^B^ compared to the corresponding inbred background, respectively (Additional file 1: Figure S15a-c). To evaluate the effect of RegionA on the global transcriptome, DEGs were filtered according to three criteria: (1) the DEGs located within the RegionA were excluded; (2) to rule out the interference from the imperfect purity of the genetic background of the NILs, we filtered out the DEGs that contain Mo17 SNPs in B^M^ or B73 SNPs in M^B^ beyond RegionA, as well as the target genes of B73/Mo17 eQTLs [85] which overlapped with SNPs among the NILs used in this study (see “Methods”); (3) only the overlapping up- or downregulated DEGs between B^M^ and M^B^ datasets were retained. A total of 473 highly confident DEGs putatively modulated by RegionA were obtained, including 255 genes that downregulated in B^M^ and upregulated in M^B^, 143 genes that upregulated in B^M^ and downregulated in M^B^ (Additional file 1: Figure S15d). GO enrichment analysis of these DEGs revealed that they were mainly enriched in transcription and metabolic regulation response to stress, in particular, to temperature stress response (Additional file 1: Figure S15e). We further analyzed public maize landrace data from Meso- and South America (Additional file 2: Table S8) [39]. In agreement with the putative role of temperature response, we found that RegionA-absence lines predominantly enriched in the highland environment, where the general temperature is cooler (Additional file 1: Figure S16). Indeed, a significant association between RegionA and altitude was detected (F-test, *P* value = 0.0219, Additional file 2: Table S9). To test whether RegionA might be related to some agronomic traits, we performed association analysis between RegionA and phenotypes using a previously reported association panel [86]; our results suggest that RegionA was significantly associated with heading date and ear height (*P* value = 6.12E−04, 4.59E−04, respectively). In summary, these results suggest that the evolution adaptation of *Zea* family to various environments may be attributed to the phenotypic consequence caused by the megabase-scale structure variation of the RegionA.

## Discussion

### Recombination repression contributed to the preservation of large PAVs

Despite the growing evidence supporting the role of PAVs in the intraspecific genome evolution, megabase-scale PAVs have been rarely characterized in plant species including maize. It had been difficult to detect those large PAVs possibly due to the lack of long-read sequencing data until recently, but another major reason is that large PAV-induced heterozygosity in an intraspecific hybrid could be progressively shuffled by recombination during evolution [16, 20].

The fascinating intactness of the RegionA, which is highly conserved in the presence lines versus almost entirely missing in absence lines, capacitates the detection of megabase-scale PAVs as survival from evolution erosion. A strong negative association between transposable elements (TEs) and meiotic recombination rates across the genome was confirmed [87–90], while the localization of recombination also tends to show negative correlations with local repetitive-element densities [68, 91]. Consistent with that, the pericentromeric location warrants the suppressed recombination around RegionD [68], facilitating the preservation of this large PAV from convergence. RegionA, located on the distal chromosome arm end, seemed to challenge the above hypothesis (Fig. 4). This paradox was reconciled by our later finding that RegionA, located within the NOR, is flanked by rDNA. It has been found few meiotic DSBs occur in the rDNA in yeast [91]. A number of meiotic DSBs within the rDNA were reported in maize [69]; however, due to an underestimate of the abundance of rDNA copies used in their calculations, meiotic DSBs in the rDNA may actually be quite infrequent. Furthermore, suppressed recombination rate in the rDNA region has been justified [68, 70], suggesting the occasional DSBs in the rDNA tend to be repaired by any other mechanism but homologous recombination. A recent study in Arabidopsis confirmed the model that highly repetitive 45S rDNA arrays are protected from SPO11-induced meiotic DSBs as well as the meiotic recombination machinery by their recruitment into the nucleolus [92]. Our finding on the RegionA within the NOR highlighted the consequence of the diminished recombination, which led to the largest PAV found in maize showing highly conserved sequence as a “fossil” in the long term of evolution (Figs. 3 and 4). The enrichment of repeat sequences in the recombination coldspots explained the difficulty in the assembly of RegionA flanking rDNA region (Additional file 1: Figure S1c,d). This also implies that large PAVs may be underestimated, considering that most genome assemblies nowadays are not based on long-read technologies.

### Ancient “fossil” PAV unveils the *Tripsacum* segment migration into *Zea*

**Fig. 6:**
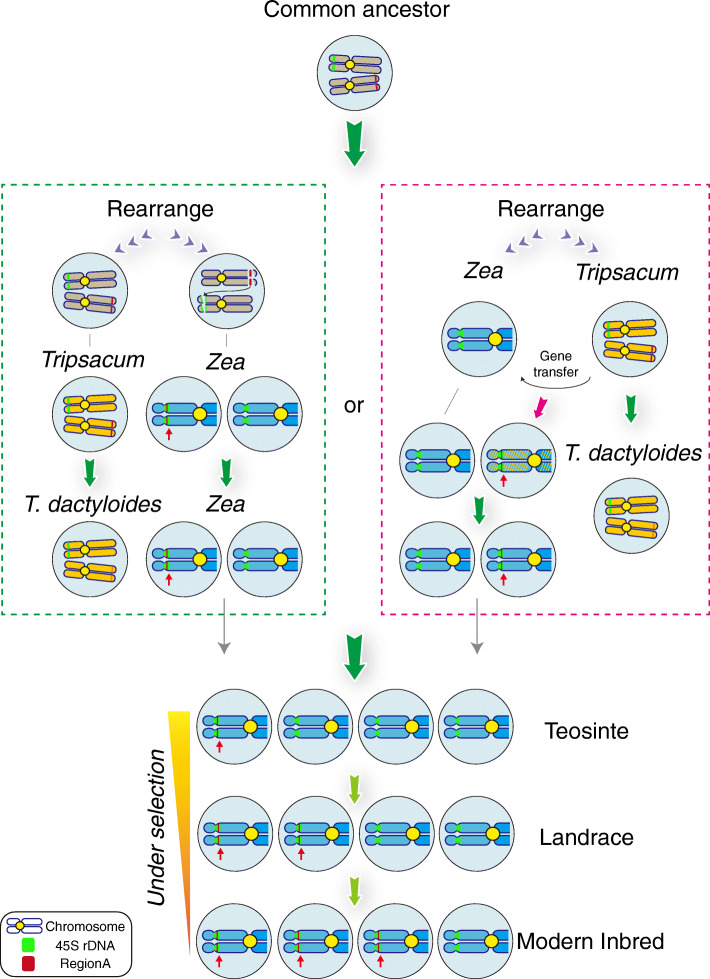
Schema chart of relationships between RegionA and 45S rDNA from divergence to domestication and improvement. Two possible scenarios for the RegionA origin were shown: **a** RegionA originated from the common ancestor of *Tripsacum* and *Zea*, and their sequence conservation resisted the reshuffling through the long term of evolution; **b** the introgression occurred during the process of *Zea* speciation, and at that moment *Tripsacum* could possibly be compatible to cross with. For *Zea* genus (blue chromosomes), RegionA fragments (red) were always protected by rDNA (green); for *Tripsacum* (yellow chromosomes) and common ancestor (gray chromosomes), RegionA fragment (red) and rDNA (green) were located in different chromosomes

Nowadays overwhelming evidence from genomics and molecular biological analysis authentically proved maize domestication from its wild ancestor teosinte. However, the previous process, speciation of *Zea*, remains to be elucidated. Along with the speciation raised by interspecific hybridization, a simultaneous effect is that the homologous recombination and introgression between ancestral species also generate PAV in the genome [29–31]. Thanks to the integrity of the RegionA protected by the NOR, providing the opportunity to trace the footprint may be preserved against the evolution dynamic. RegionA was found conservatively shared among *Tripsacum* and *Zea*, but not detectable in more distant sister species, suggesting a close involvement of *Tripsacum* in the *Zea* evolution. Our in-depth analysis revealed the drastically reduced absolute genetic divergence between *Tripsacum* and *Zea* at RegionA compared with other loci in the genome (Additional file 1: Figure S9), which was generally considered as the sign of recent gene flow distinguish from shared ancestral variation [66, 67]. We also found evidence supporting gene migration from *Tripsacum* to teosinte and maize landrace (Fig. 2), raising our postulation that the ancient *Tripsacum* might contribute to *Zea* speciation. The flow signal could be only detected from RegionA but not any other four large PAVs reported in this study or any location from the genome. In addition to teosinte parviglumis, RegionA is also stably present in other *Zea* genus as detected by oligo-FISH in *Zea nicaraguensis*, *Zea luxurians*, *Zea mays* ssp. *huehuetenangensis*, and *Zea mays* ssp. *mexicana*. In all tested *Zea* samples, the location of RegionA appeared overlapped with the NOR signal, indicating the segment migration occurred no later than the divergence within *Zea* family (Additional file 1: Figure S17). Overall, our data proposed two possible scenarios for the RegionA origin: RegionA originated from the common ancestor of *Tripsacum* and *Zea*; or a direct introgression occurred from speciated ancient *Tripsacum* to speciating *Zea* when there was no intercross barrier between yet (Fig. 6). Nevertheless, in the former scenario, as NOR preserves the maize RegionA, factors underpinning the sequence conservation in *Tripsacum* are also warranted. However, the same strategy on recombination suppression by NOR can be ruled out in *Tripsacum* since the homologous RegionA localizes separately with NOR (Additional file 1: Figure S17). In contrast, the latter scenario seems more promising. This is not merely because of the more recent timing in favor of less variation, but more importantly, RegionA was enriched with *Tripsacum*-specific LTR retrotransposons, suggesting the introgression happened after the *Tripsacum*-type LTR retrotransposons were specifically expanded in this genus.

### RegionA is associated with environmental adaptation in the maize adaptation during domestication

In animals, intraspecific genomic diversity is generally thought to derive from relatively small-size variants, resulting from the selection pressure for genome integrity and biological function that otherwise could be dampened by large-size structural variations. Large-size PAVs have been occasionally described in plants, but the number of genes within them is usually limited [32, 33, 93–98]. A surprising fact of our finding here is that 70 genes are located within RegionA, and this is comparable to the average gene density in the whole genome (21.5 genes per megabase compared to a genome-wide average of 18.7 genes per megabase) [52]. In addition, these genes are actively expressed, with a dosage effect between presence and absence lines, linking a possible phenotypic variation to be further investigated as an inset understanding of the heterosis.

Since unbalanced PAVs are present only in some individuals, they have been referred to as dispensable elements generally unnecessary for survival. However, growing evidence supports their roles in various biological processes in plants, arguing that genes within PAVs may be significant in special conditions or particular individuals [16]. Functions of abiotic/biotic response to environmental stresses have been found in PAVs from multiple plant species, and traits of stress response were complementary between different genomes [16, 33, 93–95, 97–100]. More specifically, similar examples of maize SVs have been found to be causative to diverse phenotypes [101–103]. Indeed, a 14 Mb large inversion (*Inv4m*) was previously identified as introgression from *mexicana* to high elevation landraces, which is also associated with highland environment adaptation and earlier flowering times in highland maize [103]. Consistent with that, we found the transcriptome effect from the largest B73/Mo17 PAV was enriched in GO categories of the stress response, particularly related to the temperature responding pathway (Additional file 1: Figure S15e). We also observed that RegionA is predominantly absent from the highland landraces (Additional file 1: Figure S16d), implying the role of RegionA functions to modulate the adaptive growth in the tropical lowland, whereas being a fitness cost for the cold environment individuals in the highland.

It is worth noting that the RegionA regulatory function on heat response may not be the only cause for the strong selection signal (Fig. 5), since genes attributed to beneficial agriculture traits could also be the selection targets. Our association analysis suggests that RegionA was related to heading date and ear height. Moreover, a major QTL for grain yield (GY) has been recently reported accounting for 13% of the variance in GY, this 17 Mb QTL interval coincided with RegionA, and Mo17 allele as one of the founders for the mapping population contribute to low GY values [104]. Another RegionA located gene, *Scmv1*, encoding atypical h-type thioredoxin, has been identified to impart strong resistance to Sugarcane mosaic virus (SCMV) [105]. In addition, a significant SNP for maize upper leaf angle was found located in RegionA [106]. These data suggest the *Tripsacum*-originated regions indeed play critical roles in genome evolution of *Zea*, as well as the complex interplay between the genome and the environment.

Intriguingly, the contribution of archaic introgression to modern maize is not unique in plants, but there has been increasing evidence showing that introgression from Neanderthals and Denisovans to the human ancestors was subject to selection for the genetic architecture of adaptive traits [107–110]. In general, the introgression from Neanderthals functions as deleterious variations thus have been under negative selection during the evolution [107, 111]. In contrast, a hypoxia pathway gene, *EPAS1*, was previously identified with positive selection in Tibetans for the altitude adaptation. The *EPAS1* haplotype from Tibetans, associated with optimal hemoglobin concentration for high altitude, was found to be introduced by an admixture from Denisovan [110, 112]. Together with these findings, our study highlights a convergent approach during the genome evolution between plant and human, resulting from the adaptive nature of archaic introgression.

## Conclusions

In this study, a 3.2-Mb PAV element was characterized with high sequence integrity in maize diversity populations, and the embedded location in the nucleolus organizer region (NOR) was associated with the suppressed recombination and preserved the sequence footprint against the evolutionary variation. A series of analyses reveal the striking genomic footprints from *Tripsacum* in the maize genome, unveiling the origin of this PAV of *Zea* genus from *Tripsacum*. We also found this *Tripascum*-originated segment was under selection during the *Zea* domestication and adaptation, which is consistent with its role for environmental fitness and agronomic traits. In a general perspective, our results instantiates a strategy for evolution and function analysis of the “fossil” structure variations during genome evolution and speciation, providing a neglected view by the published genome-wide PAV studies.

## Methods

### Plant materials

For transcriptome sequencing, the original B^M^ (B73-NIL) and M^B^ (Mo17-NIL) lines MBNIL_b131 and MBNIL_m065 developed by Eichten et al. [84] were requested from Maize Genetics Cooperation Stock Center, backcrossed with B73 and Mo17 respectively for two generations, followed by one round of self-pollination. Then, homozygous RegionA-absence seeds were selected as B^M^, and homozygous RegionA-presence seeds were selected as M^B^. Accessions of other germplasm used in this study were listed in Additional file 2: Table S10.

### Generation of B73 AGPv4.1 genome

An updated B73 Chr 6 assembly was kindly provided by Dr. Yingping Jiao at Doreen Ware Lab in the Cold Spring Harbor Laboratory. We replaced the original Chr 6 in AGPv4 with this new Chr 6, and named this new genome assembly AGPv4.1. B73 Hi-C data were mapped to the maize genome AGPv4 and v4.1 respectively using Bowtie2 [113], then detected the valid contacts and generated iterative correction matrixes using HiC-Pro v2.10.0 [114], contact matrix was visualized by Juicerbox 1.8.8 [115].

### Presence absence region identification

Raw reads were quality trimmed using Trimmomatic v0.38 [116] with the following parameters (LEADING:3, TRAILING:3, SLIDINGWINDOW:4:15, MINLEN:36). Then the trimmed reads were aligned against the B73 AGPv4.1 genome using Bwa mem [117]. Reads with high mapping qualities (MAPQ ≧ 30) were retained for downstream analysis. Read depth was calculated in 10-kb windows with step size of 1 kb (at least one base pair overlapped). The windows containing less than 10 reads per genome coverage were defined as absence region.

### RegionA genotype identification

Copy number variation analyses were conducted to identify the copy number of RegionA by cnv-seq [118], using the maize natural variation population (100 HapMap2 line and 99 Meso- and South America landrace) as treats, B73 as a reference, with 100-kb windows. K-means clustering analyses were performed for each line according to their adjusted copy number ratio (CNV ratio) of RegionA windows. When *k* = 3, we can easily distinguish three genotypes of RegionA: Homozygous absence (0/0), the log_2_ of CNV ratio ranges from − 2~− 4; Homozygous presence (1/1), the log_2_ of CNV ratio are approximal 0.6~0.7; Heterozygous (0/1), the log_2_ of CNV ratio are approximal − 0.3, which showed about half of CNV ratio relative to RegionA-presence homozygous. Although the log_2_ of CNV ratio of Homozygous presence (1/1) genotypes were slightly higher than the whole genome (0.6 versus 0), we think it may be due to the extremely low genetic diversity of RegionA, so that RegionA contained more reads with high-quality alignments than the rest of the genome.

### Oligo probe design

The oligo probe used in this study was designed following a previously published protocol [119]. Briefly, after removing repetitive sequences, RegionA was divided into oligos (59 nt) with a step size of 5 nt. Oligos were then mapped to maize genome and those mapped onto (> 75% homology over all 59 nt) multiple loci in the genome were discarded. Next, the melting temperature Tm and hairpin Tm of each oligo sequence were calculated, and oligos with dTM > 10 (dTM = melting temperature − hairpin Tm) were retained. Oligos were synthesized by MYcroarray (Ann Arbor, MI, USA) and labeled with digoxigenin, following a previously published protocol [119]. Oligo probe information is listed in Additional file 2: Table S11.

### FISH

Kernels of maize and *Zea* genus were germinated in a growth chamber for 2–3 days, and 1–2 cm primary root tips were collected for FISH analysis. Mitotic metaphase and pachytene chromosomes were prepared as described previously [120]. FISH and oligo-FISH were performed according to previously published protocols [121, 122] . Briefly, DNA probes were labeled with biotin-11-dUTP (Vector Laboratories) and digoxigenin-11-dUTP (Roche) via nick translation. Chromosomes were counterstained with DAPI in VectaShield antifade solution (Vector Laboratories). Biotin- and digoxigenin-labeled probes were detected by anti-biotin fluorescein (Vector Laboratories) and anti-digoxigenin rhodamine (Roche), respectively. FISH images were captured with the Olympus BX61 epifluorescence microscope equipped with a CCD camera (QImaging; RETGA-SRV FAST 1394), and processed with Image-Pro Plus 6.0 software (Media Cybernetics). Adobe Photoshop CS3 software was used for final contrast adjustment of the images. LTR-RT fragments used for FISH probes were amplified with primers listed in Additional file 2: Table S12.

### Estimation of insertion timing for LTR-RTs

We aligned the 5′ LTR and 3′ LTR of each intact LTR-RT by MUSCLE v3.8.31 [123], and then used a K2P substitution model to calculate relative ages using the R package APE [124]. We converted these relative ages to absolute time using a mutation rate of 3.3e−8 [125].

### Footprint tracing of *Tripsacum*-specific retroelement in maize

All transposable element annotation were fetched from Gramene [126] : ftp://ftp.gramene.org/pub/gramene/release-62/gff3/zea_mays/repeat_annotation/. RegionA LTR-RTs were re-annotated using previous methods [52] with B73 AGPv4.1 genome sequence. Random regions were generated by Bedtools [127] with function *random* for retrotransposon family comparison.

To identify the low abundance LTR-RTs in B73, genome-wide LTR-RTs were mapped against the 5′LTR sequences of all the RegionA LTR-RTs using BLASTN with default parameters. The results were then filtered with 80–80–80 rule [128], by which the elements show at least 80% similarity using at least 80 base pairs of 80% of the element’s 5′LTR. We calculated the hit number for each RegionA LTR-RTs. LTR-RTs with hit number less than 100 were thought as low abundance LTR-RTs in B73.

To identify the high abundance LTR-RTs in *Tripsacum*, we extracted the entire element sequence for all the RegionA LTR-RTs, then resequencing data of *Tripsacum* and *Zea luxurians* were mapped against these sequences using Bwa [117] with default parameters. To correct the differences of sequencing data depth and length of elements, reads per kilobase per million mapped (RPKM) was calculated for each LTR-RT. If a LTR-RT has a ratio of RPKM_*Tripsacum*_ to RPKM_*luxurians*_ more than 2, and the RPKM_*Tripsacum*_ was more than 30 but RPKM_*luxurians*_ was less than 30, we thought it as high abundance LTR-RT in *Tripsacum*.

The intersection of low abundance LTR-RTs in B73 and high abundance LTR-RTs in *Tripsacum* were designated as *Tripsacum*-specific enriched LTR-RTs (Tse-LTR-RTs). All genomic sequences of *Tripsacum* *dactyloides* clones (TC-25, TC-24, TC-12, TC-5, TF-B8-15, TF-B5-2, TF-B5-3) [64] were downloaded from NCBI, then we mapped these sequences against the RegionA 5′LTR sequences from B73 AGPv4.1 using BLASTN with default parameter. After filtering the BLASTN results with the 80–80–80 rule [128], these remaining LTR-RTs were classified as *Tripsacum* clones consistent LTR-RTs.

B73 annotated CDS sequences in RegionA were extracted, and unique CDS for each gene was achieved by merging the transcripts for the genes with multiple transcript isoforms. *Tripsacum* resequencing reads were aligned to B73 genome and the percentage of CDS coverage was calculated using in-house Shell scripts. A *Tripsacum* consistent gene was defined by coverage higher than 70%.

### Genetic introgression identification

Due to the low coverage of the previously accessible resequencing data from *Tripsacum*, resequencing of three additional *Tripsacum* lines was conducted, generating coverage depth ranging from 11.22× to 14.48×. In addition to four *Tripsacum* lines, RegionA-presence lines from the diversity panel were retained for the following analysis, including teosinte (*n* = 5), landrace (*n* = 11), and inbred (*n* = 50). Allele frequencies for each subgroup were estimated using the ANGSD “-doMafs” command [129]. Only polymorphic sites for which 50% of the individuals in each group have read support were retained. Introgression events among *Tripsacum*, teosinte, maize landrace, and modern inbred were detected by Treemix [130]. For each number of migrations, the maximum likelihood (ML) tree was generated based on 1000 replicates with a block size of 5000 sites.

We used the absolute sequence divergence (*d*_*xy*_) to examine genetic differentiation across the genome between *Tripsacum* and *Zea*, only RegionA-presence lines were used for calculating, *d*_*xy*_ was computed using the script popgenWindows.py (github.com/simonhmartin/genomics_general) with a sliding window of 20 kb and a step of 10 kb.

### Transcriptome and histone modification data analysis

For the transcriptome analysis of the NIL lines, B^M^, M^B^, B73, and Mo17 plants were grown in a growth chamber at 28 °C for 12 h of light and 25 °C for 12 h of darkness for 2 weeks before harvested for RNA extraction. RNA was isolated using a Quick RNA isolation Kit (Huayueyang Biotechnology of Beijing, http://www.huayueyang.com/), followed by mRNA library construction (Novogene) and sequencing at Illumina HiSeq2500 platform.

RNA-seq reads were mapped to B73 AGPv4 genome by HISAT2 [131]. Cufflinks v2.2.1 [132] was used to estimate normalized gene expression values (FPKM). Differential expression analysis was carried by Cuffdiff v2.2.1 [133]. Genes with an expression fold change ≧ 2 and FDR ≦ 0.05 were defined as differentially expressed. Comparison between B^M^ and B73, as well as between M^B^ and Mo17, were conducted for the differentially expressed genes (DEGs) of B^M^ and M^B^. The overlapped DEGs of B^M^ and M^B^ were used for downstream analysis. We merged bam files of replicates respectively and discarded the low-quality reads (MAPQ < 20), then all samples were conducted joint-calling by samtools mpileup [134] and sites with QUAL < 10 and DP < 5 were discarded. The DEGs that contain Mo17 SNPs in B^M^ or B73 SNPs in M^B^ were filtered out. The B73/Mo17 RIL population eQTLs that overlapped with SNPs between B^M^ and B73 or M^B^ and Mo17 (a 1-kb window centered on top SNPs in the eQTLs) were extracted [85]; the target genes of these overlapped eQTLs were also filtered out. The Singular Enrichment Analysis (SEA) tool in AGRIGO 2.0 [135] was applied for GO enrichment analysis of the final gene set, with default parameters.

The analysis of the transcriptome atlas datasets for tissue types was conducted through the same RNA-seq workflow as described above. We applied a more stringent criterion for identifying B73-specific PAV genes to Mo17. First, we identified a total of 3723 B73 singleton genes using the Genetribe pipeline [136]. Then, we filtered those with more than 30% CDS regions covered by Mo17 resequencing reads. Finally, we obtained 911 B73-specific PAV genes, 43 of which locate in RegionA.

We calculated the gene expression and made comparisons among B73/Mo17 hybrids and B73 for different tissues, only expressed genes (FPKM ≧ 1 in at least one sample) were retained. For tissues consisting of diploid cells, B73-specific genes showing a log_2_fc > 0 in hybrid than B73 were classified as non-additive (higher) genes, while those showing log_2_fc < − 2 were classified as non-additive (lower) genes. For endosperm (triploid cells), B73-specific genes showing log_2_fc_(BM/B73)_ > log_2_(2/3) + 1 or log_2_fc_(MB/B73)_ > log_2_(1/3) + 1 were classified as non-additive (higher) genes, while those showing log_2_fc_(BM/B73)_ < log_2_(2/3)− 1 or log_2_fc_(MB/B73)_ < log_2_(1/3)− 1 were classified as non-additive (lower) genes.

ChIP-seq data were mapped to the B73 AGPv4 reference genome using Bowtie [137], and the average enrichment levels of transcription start site (TSS) ± 2 k bp and transcription termination site (TTS) ± 2 k bp for RegionA, RegionB, and RegionC genes (FPKM ≧ 1) were calculated respectively.

### Data uplifting from AGPv4 to AGPv4.1

The HapMap3 genotype data [54] and ZeaGBSv2.7 data are available at http://cbsusrv04.tc.cornell.edu/users/panzea/filegateway.aspx?category=Genotypes. The sequence of upstream 150 bp to downstream 150 bp around these SNPs was extracted and performed BLASTN against the AGPv4.1 genome, only unique alignment of length = 300 with no mismatch were retained. The locus of filtered SNPs was transformed to the new position where these sequences aligned.

### Population genetics analysis and selection test

SNPs were pre-processed for the following analysis, for which, SNPs with a missing rate of more than 50% were discarded. To construct the phylogenetic tree, we extracted the uplifted HapMap3 SNPs of upstream 100 kb and downstream 500 kb of NOR region in chromosome 6, with a missing rate below 0.5 and a minor allele frequency (MAF) above 0.05 among the 100 HapMap lines; for each line, missing rate more than 0.5 was discarded. Finally, total 90 lines were used for the neighbor-joining tree construction by MEGA 7.0.26 [138] under the *p-distances* model with bootstrapping (1000), and sites with gaps or missing data more than 20% were deleted. Ggtree [139] was used to visualize the tree.

For nucleotide diversity (π), *θ*_*W*_ [140] and Tajima’s *D* [141], we divided the teosintes, landraces, and inbreds into 6 groups (teosintes-presence, teosintes-absence, landraces-presence, landraces-absence, inbreds-presence, inbreds-absence) according to RegionA’s presence or absence, then we calculated nucleotide diversity (π), *θ*_*W*_, and Tajima’s *D* for these groups respectively using Vcftools 0.1.15 [142] with non-overlapping windows of 10 kb size.

SweeD [74] was used for detecting complete selective sweeps in the landraces and modern inbreds, and the composite likelihood ratio (CLR) was calculated with grid sizes of 50 kb across each chromosome. We then determined the significance threshold to reject the null model of neutrality based on the demography of maize domestication [75]. More specifically, this demographic model estimates an ancestral effective population size of *N*_*a*_ about 123,000, and maize split from teosinte about 15,000 generations ago with an initial size of about 5% of the ancestral *N*_a_; maize experienced an exponential population growth after its split from teosinte, reaching a final modern effective population size of 2.98*N*_a_. The gene flow from teosinte to maize was estimated to be *M*_tm_ = 1.1 × 10^−5^ × *N*_a_ migrants per generation, from maize to teosinte was estimated to be *M*_mt_ = 1.4 × 10^−5^ × *N*_a_ migrants per generation. The mutation rate per year (*μ*) used here is 3e−8, assuming no recombination. In fact, the effective population size (*N*_e_) in maize is difficult to estimate because of rapid demographic change during and post domestication; here, we ran the models with a set of different *N*_e_ values (100 k, 500 k, and 1 M). We used Hudson’s ms [147] to perform coalescent simulations and 10,000 1-Mb simulation datasets were generated, and the simulation datasets were used as inputs for SweeD. The highest 99.99th percentile of the CLR distribution of simulation datasets was used as a significance threshold for real data (CLR = 4.971903, 5.053264 and 5.127507 for *N*_e_ values = 100 k, 500 k, and 1 M). For modern inbreds, which are different from a population at equilibrium, we considered genomic regions with the top 5% of CLR scores as a significance threshold (CLR = 6.258513).

### Profiling of the recombination landscape

Uplifted ZeaGBSv2.7 data were used to identify recombination events. We extracted US-NAM population [68] GBS data that consists of 5000 F6 RILs from raw data, and divided them according to 25 diverse inbred founders. First, we converted SNPs of HapMap format to abh format using Tassel 5.2.40 [143], which filtered the missing loci, heterozygous loci, and sites with same genotypes in parents. Then, we constructed bin-map with 20 SNP windows and a sliding step of 2 SNPs, in each window, and the genotype was defined by the ratio of two kinds of parent genotypes: if the proportion of one kind of genotype in this window is more than 3/4, this kind of genotype will be called, whereas if none of genotype is more than 3/4, the window will be called heterozygous genotype. Regions with no more than ten continuous heterozygous windows were set as breakpoints. We merged the crossover events of presence versus absence (P-vs-A) population and presence versus presence (P-vs-P) population respectively. For chromosome 6 of AGPv4.1, we calculated the crossovers counts in 1-Mb non-overlapping windows across the whole chromosome. For P-vs-P population, counts in each window were divided by 3786 which is the size of P-vs-P population. While for P-vs-A population, counts in each window were divided by 1198 which is the size of P-vs-A population.

### Association analysis of RegionA presence/absence and altitude

The whole-genome shotgun sequencing data and geographic information were downloaded from Figshare https://figshare.com/articles/GenomeSize_lowcoverage_Maizedata/5117827 [39]; after discarding extremely low coverage data (< 0.01× coverage) and non-geographic information data, we fetched a total of 80 landrace accessions. To identify the presence or absence of RegionA, these data were mapped against B73 AGPv4.1 genome by Bwa mem [117], high-quality reads (MAPQ ≧ 30) were retained, and read depth was calculated in 10-kb windows with step size of 1 kb. For each window, read depth was normalized with the total read number for each sample. Heatmap was generated with R package *pheatmap* [144]; all columns were clustered with hierarchical clustering method according to normalized read depth. PCA was performed using Tassel 5.2.40 [143] with default settings. An F-test was used to test the association between RegionA and the altitude, by comparing a full model (altitude ~ PC1 + PC2 + PC3 + PC4 + PC5 + Genotype) and a reduced model (altitude ~ PC1 + PC2 + PC3 + PC4 + PC5), with the “anova” and “lm” package in R (Supplemental Table 10).

### Association analysis of RegionA presence/absence and agronomic traits

To test the association between RegionA and agronomic traits, we used the maize association population previously reported [86]; two pairs of primers designed within RegionA were used to determine RegionA presence/absence. Phenotypic data of association population were fetched from http://www.maizego.org/Resources.html; the associations between RegionA and phenotypes were tested using a mixed linear model that corrects for population structure and family relatedness [145] in Tassel 5.2.40 [143].

## Supplementary Information

**Additional file 1: Figure S1.** Hi-C interaction matrix near representative PAVs with different B73 genome assembly version. **Figure S2.** Genotype variation and percentage of presence bin among different lines in the other representative B73 PVs. **Figure S3.** Genotype variation and percentage of presence bin among different lines in the other representative Mo17 PVs. **Figure S4.** Syntenic dotplot between B73 AGPv4 (Chr6) and genomes of PH207, B104, Mo17, W22, 24 NAM founders and some other outgroup species (rice and setaria). **Figure S5.** The majority of RegionA genes are either completely present or absent in diversity lines. **Figure S6.** RegionA genotypes in the diversity panel of maize. **Figure S7.** Characteristics of RegionA transposable elements. **Figure S8.** Gene flow detected among *Tripsacum* and *Zea* for (a-b) RegionA when (a) m=2 and (b) m=3; for (c-e) maize whole chromosome 6 when (c) m=1, (d) m=2 and (e) m=3. **Figure S9.** Absolute sequence divergence (*d*_xy_) between *Tripsacum* and *Zea*. **Figure S10.** Genetic diversity and selection signals among different populations. **Figure S11.** Transcription profiling of the genes in PAVs by RNA-seq for (a-c) diploid cells and (d-f) triploid cells. **Figure S12.** Histone modification change with PAVs genes in (a-i) seedling shoot (diploid cells) and (j-m) 12 DAP endosperm (triploid cells) dissected from B73, Mo17 and the reciprocal hybrids. **Figure S13.** Venn diagram of intersections of non-additive RegionA genes in different tissues. **Figure S14.** Schematic diagram shows genotype of the B73-Mo17 Near-Isogenic Lines (NILs) and their parental inbreds selected for transcriptome sequencing. **Figure S15.** Transcriptomic response resulting from the RegionA PAV on the genome background. **Figure S16.** Presence or absence of RegionA was significantly associated with altitude of maize landrace. **Figure S17.** FISH analysis of the relative localization of the RegionA (red signal, marked as Oligo) and 45S rDNA (green signal, marked as 45S) on metaphase chromosomes of *Zea* genus and *Tripsacum*.

**Additional file 2: Table S1.** Summary of representative PAVs in B73 and Mo17 genome studied in this research. **Table S2.** List of lines used to characterize PAVs in this study. **Table S3.** Tripsacum-specific LTR-RTs in RegionA. **Table S4.** Collection of data used to examine the dosage effect of the B73/Mo17 PAVs. **Table S5.** Number of RegionA genes showing non-additive mode. **Table S6.** Details of non-additive RegionA genes. **Table S7.** Enrichment analysis of RegionA genes. **Table S8.** Geographic information of the landraces used in this study. **Table S9.** Association between the presence/absence of RegionA and altitude, tested by an F-test. **Table S10.** A summary of plant samples. **Table S11.** RegionA-specific oligo FISH probe used in this study. **Table S12.** Primers used in this study.

**Additional file 3.** Review history.

## Data Availability

The genomic resequencing data have been deposited into NCBI BioProject under accession number PRJNA639380 [146]. RNA-seq can be downloaded from NCBI SRA database with accession number SRP251136 [147].
