# Supplementary material for: Megabase-scale presence-absence variation with Tripsacum origin was under selection during maize domestication and adaptation
Source: Genome Biol. 2021 Aug 20;22:237. doi: 10.1186/s13059-021-02448-2 (PMC8377971; doi:10.1186/s13059-021-02448-2)
Supplement: Supplementary file 1 — Additional file 1: Figure S1. Hi-C interaction matrix near representative PAVs with different B73 genome assembly version. Figure S2. Genotype variation and percentage of presence bin among different lines in the other representative B73 PVs. Figure S3. Genotype variation and percentage of presence bin among different lines in the other representative Mo17 PVs. Figure S4. Syntenic dotplot between B73 AGPv4 (Chr6) and genomes of PH207, B104, Mo17, W22, 24 NAM founders and some other outgroup species (rice and setaria). Figure S5. The majority of RegionA genes are either completely present or absent in diversity lines. Figure S6. RegionA genotypes in the diversity panel of maize. Figure S7. Characteristics of RegionA transposable elements. Figure S8. Gene flow detected among Tripsacum and Zea for (a-b) RegionA when (a) m=2 and (b) m=3; for (c-e) maize whole chromosome 6 when (c) m=1, (d) m=2 and (e) m=3. Figure S9. Absolute sequence divergence (dxy) between Tripsacum and Zea. Figure S10. Genetic diversity and selection signals among different populations. Figure S11. Transcription profiling of the genes in PAVs by RNA-seq for (a-c) diploid cells and (d-f) triploid cells. Figure S12. Histone modification change with PAVs genes in (a-i) seedling shoot (diploid cells) and (j-m) 12 DAP endosperm (triploid cells) dissected from B73, Mo17 and the reciprocal hybrids. Figure S13. Venn diagram of intersections of non-additive RegionA genes in different tissues. Figure S14. Schematic diagram shows genotype of the B73-Mo17 Near-Isogenic Lines (NILs) and their parental inbreds selected for transcriptome sequencing. Figure S15. Transcriptomic response resulting from the RegionA PAV on the genome background. Figure S16. Presence or absence of RegionA was significantly associated with altitude of maize landrace. Figure S17. FISH analysis of the relative localization of the RegionA (red signal, marked as Oligo) and 45S rDNA (green signal, marked as 45S) on metaphase chromosomes of Ze [file 13059_2021_2448_MOESM1_ESM.pdf]

Megabase-scale presence-absence variation with *Tripsacum* origin was under selection during maize domestication and adaptation

Yumin Huang[1, #], Wei Huang[1, #], Zhuang Meng[2], Guilherme Tomaz Braz[3], Yunfei Li[1], Kai Wang[2], Hai Wang[1], Jinsheng Lai[1], Jiming Jiang[3], Zhaobin Dong[1,*], Weiwei Jin[1,*]

[1.] State Key Laboratory of Plant Physiology and Biochemistry, National Maize Improvement Center, Key Laboratory of Crop Heterosis and Utilization (MOE), Joint Laboratory for International Cooperation in Crop Molecular Breeding (MOE), China Agricultural University, Beijing 100193, China

[2.] Key Laboratory of Genetics, Breeding and Multiple Utilization of Corps (MOE), Fujian Agriculture and Forestry University, Fuzhou 350002, Fujian, China

[3.] Department of Plant Biology, Department of Horticulture, Michigan State University, East Lansing, MI, 48824, USA.

[#] These authors contributed equally to this work.

* To whom correspondence may be addressed. Email: zbdong@cau.edu.cn or weiweijin@cau.edu.cn

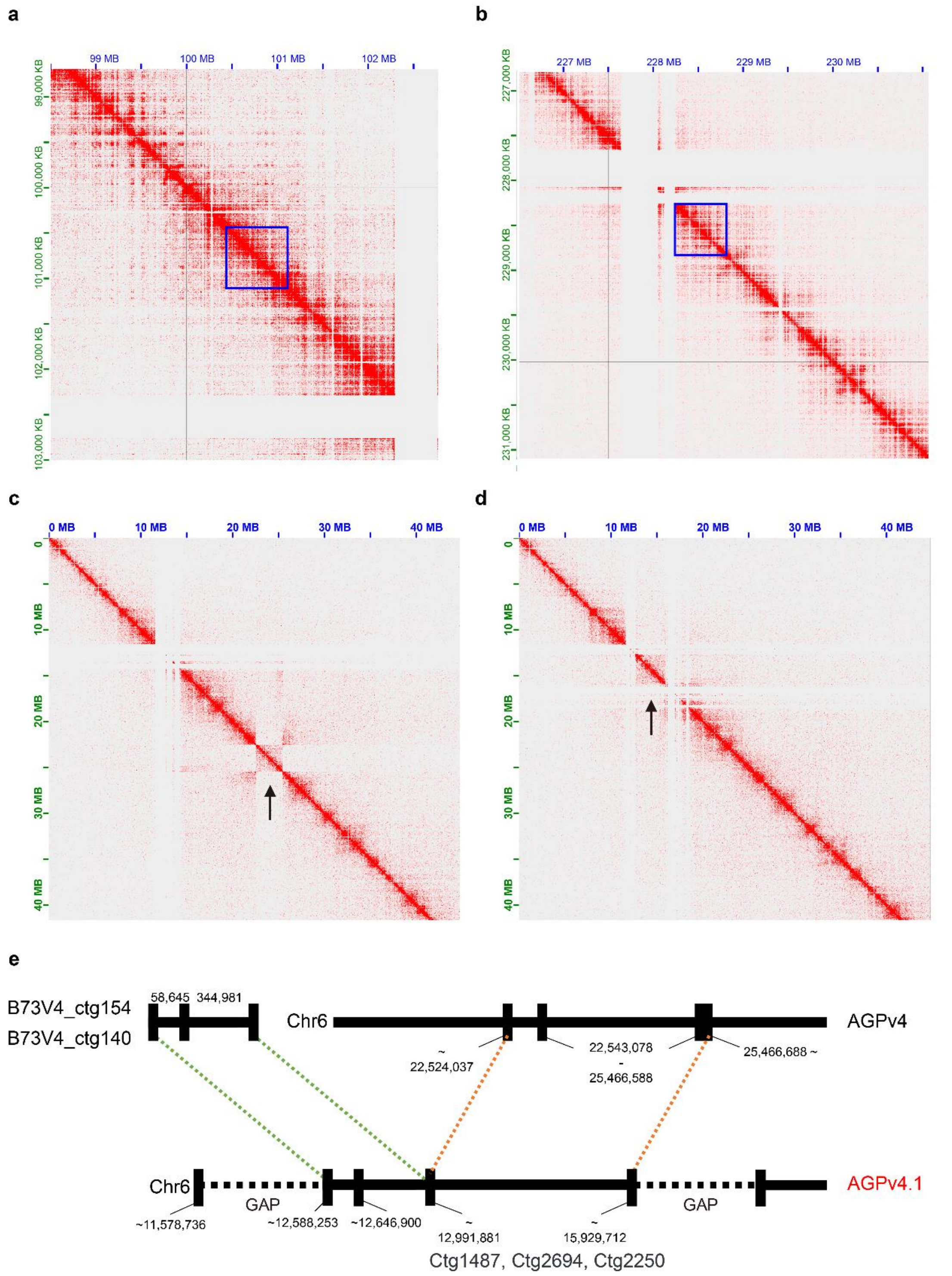

Figure S1. Hi-C interaction matrix near representative PAVs with different B73 genome assembly version: (a) RegionB in AGPv4 (RegionB indicated by blue square); (b) RegionC in AGPv4 (RegionC indicated by blue square); (c) RegionA in AGPv4, (d) RegionA in AGPv4.1 (RegionA indicated by black arrows), and (e) their correspondence.

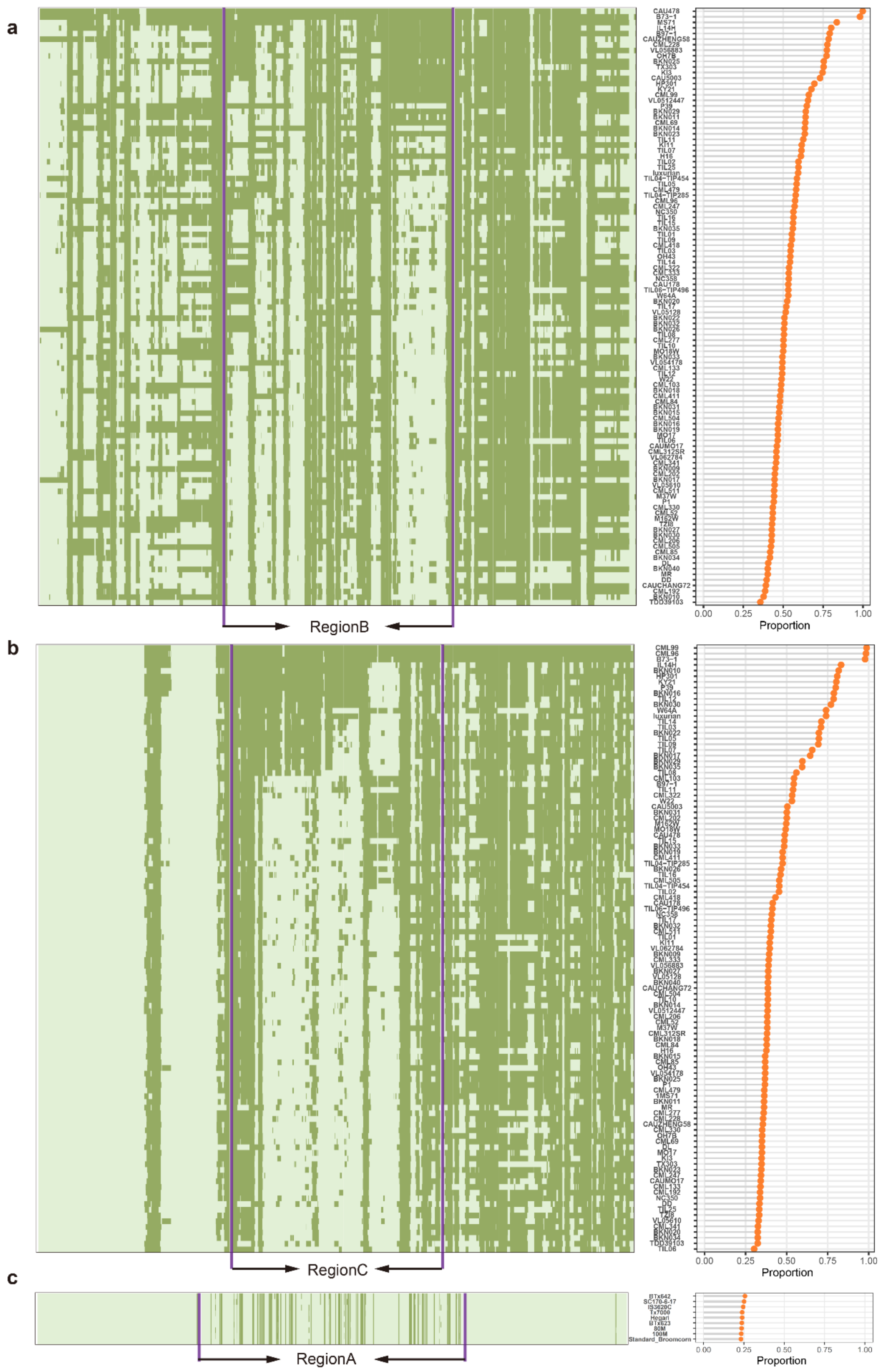

Figure S2. Genotype variation and percentage of presence bin among different lines in the other representative B73 PVs. (a) Genotype of RegionB and its flanking region (±500 kb) in hapmap2 lines. (b) Genotype of RegionC and its flanking region (±500 kb) in hapmap2 lines. (c) Genotype of RegionA and its flanking region (±2 Mb) in sorghums.

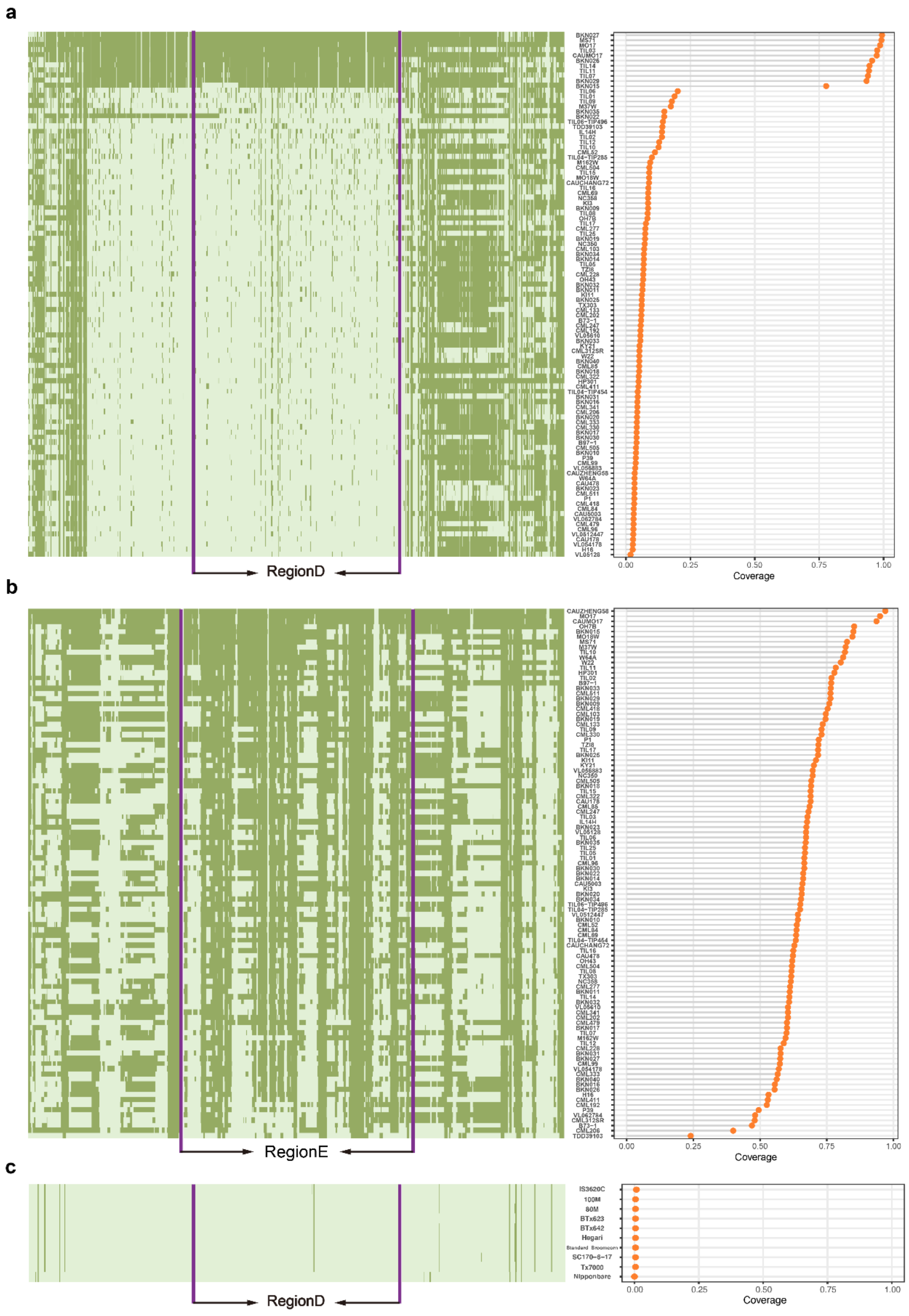

Figure S3. Genotype variation and percentage of presence bin among different lines in the other representative Mo17 PVs. (a) Genotype of RegionD and its flanking region (±2 Mb) in hapmap2 lines. (b) Genotype of RegionE and its flanking region (±500 kb) in hapmap2 lines. (c) Genotype of RegionD and its flanking region (±2 Mb) in some other species (9 sorghum and 1 rice).

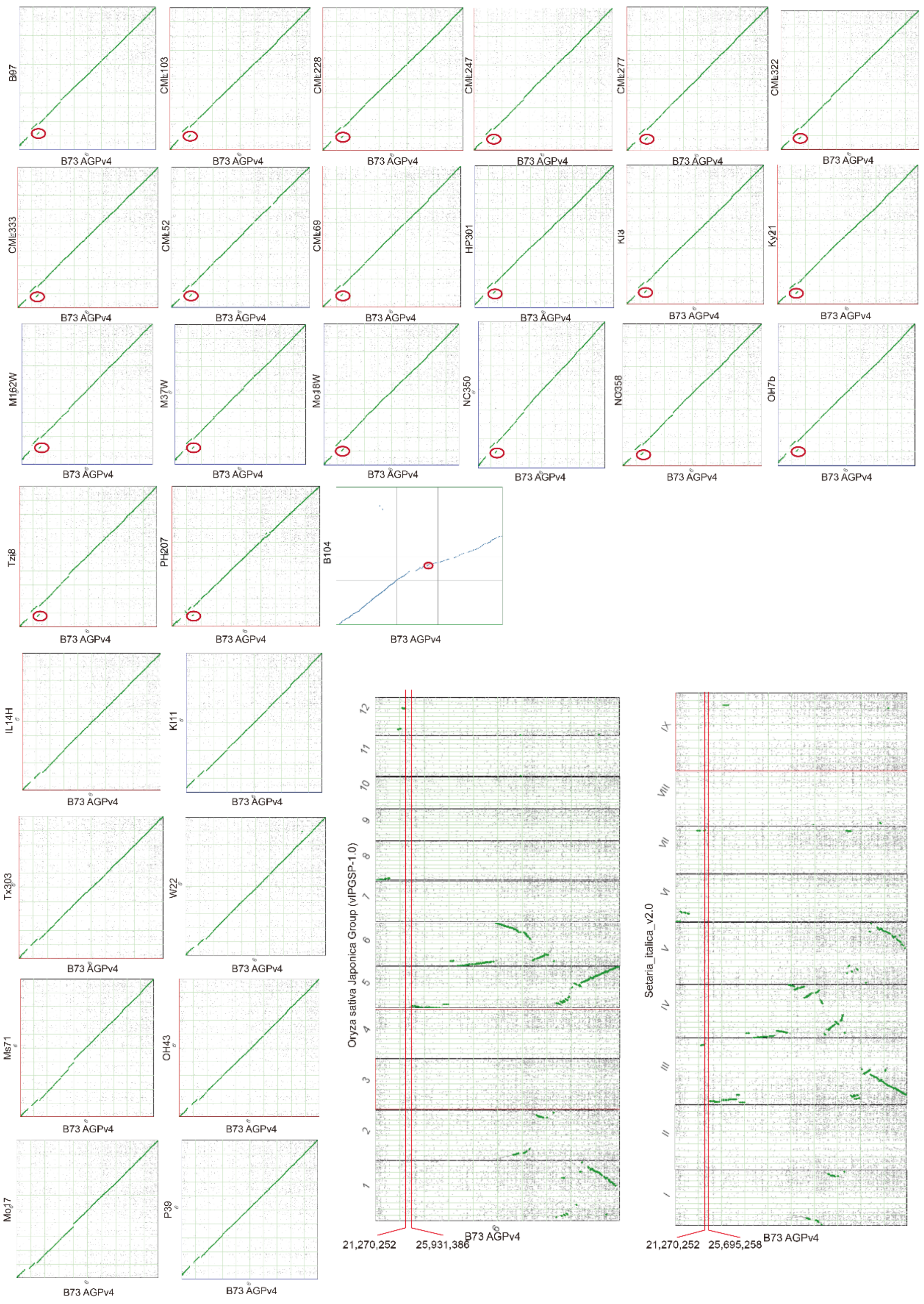

Figure S4. Syntenic dotplot between B73 AGPv4 (Chr6) and genomes of PH207, B104, Mo17, W22, 24 NAM founders and some other outgroup species (rice and setaria). Each dot represents homology genes between two genomes. Red circles indicated the collinear region.

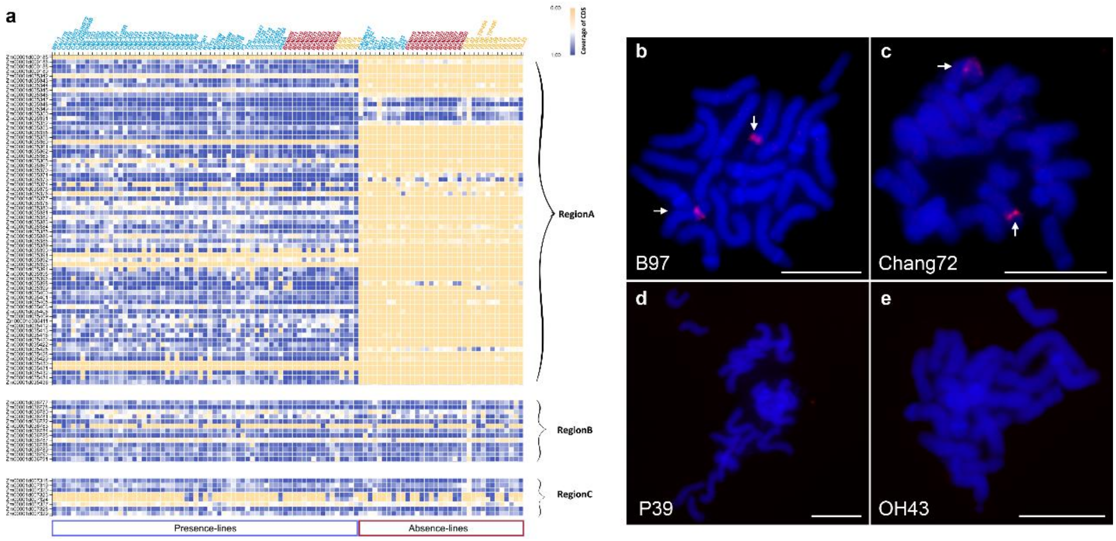

Figure S5. The majority of RegionA genes are either completely present or absent in diversity lines. (a) Coverage distribution of genes within 3 largest B73 PAV in HapMap2 lines. For each gene, the coverage of CDS region was shown, columns in the heatmap show HapMap2 lines and the rows represent genes located in these PAVs. (b-e) FISH validation using RegionA-specific oligo probes in selected maize inbred lines. RegionA-specific signals (red) were observed on a pair of homologous chromosomes of B97 and Chang72 mitosis metaphase cells (b and c), while absent on P39 and OH43 metaphase chromosomes (d and e). Scale bars = 10μm.

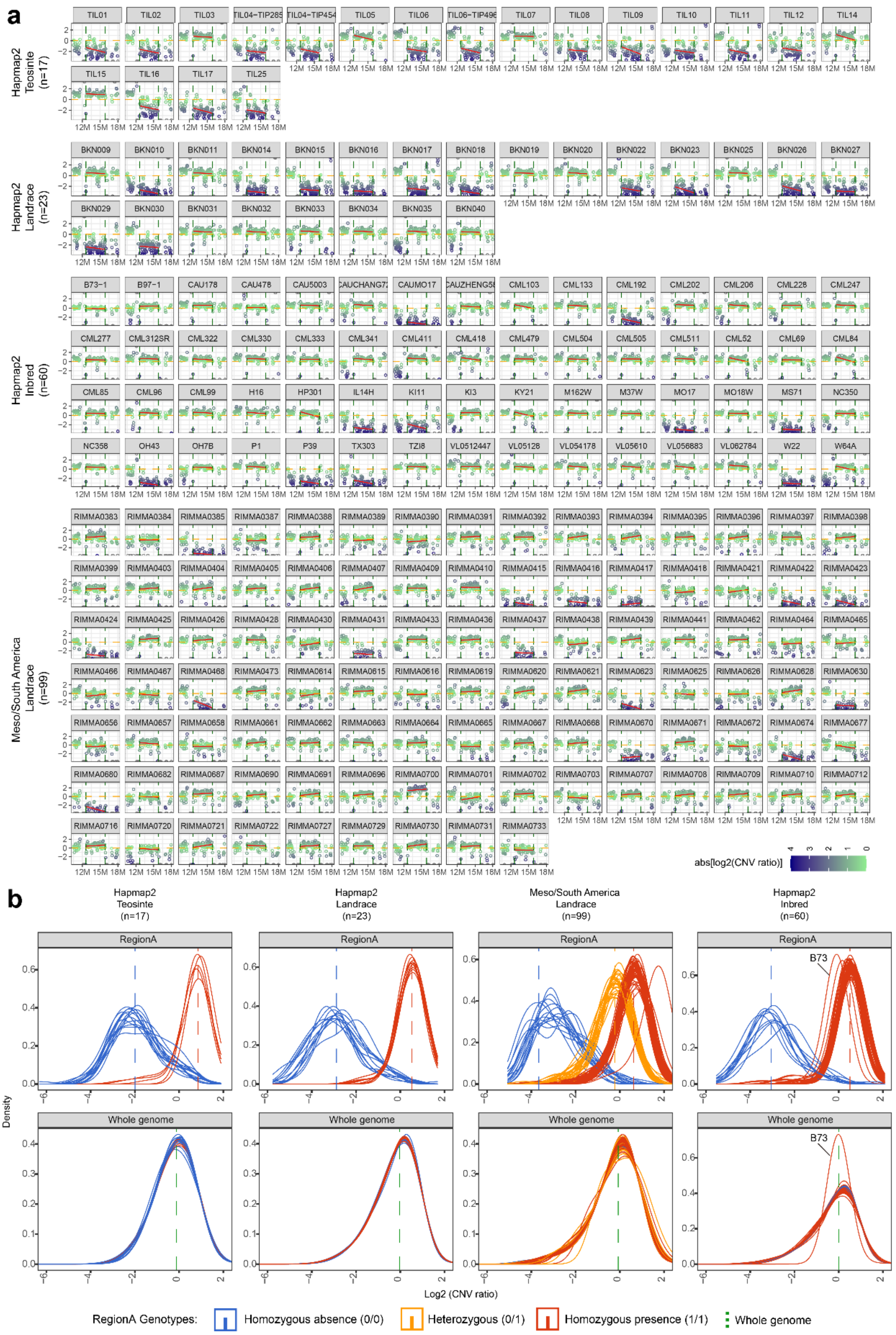

Figure S6. RegionA genotypes in the diversity panel of maize. (a) Dot plot of log2(CNV ratio) for each line. The y axis is the log2(Treat/B73 read numbers), x axis is the scale of chromosome 6 from 10M to 18M, RegionA is indicated by two green vertical dash lines, each dot represents a 100-kb window, red line indicated the fitting curve for RegionA windows. (b) Density plot of CNV ratio for RegionA windows. Each curve represents a line,

vertical dash lines represent median value for each subgroup. The mean log2 of CNV ratio for whole genome are all arrounding 0 for each line, but for RegionA, according to the results of k-means clustering, we can easily distinguish three genotypes: Homozygous absence (0/0), marked as blue, the log2 of CNV ratio are arrounding -2 ~ -4; Heterozygous (0/1), marked as orange, the log2 of CNV ratio are arrounding -0.3; Homozygous presence (1/1), marked as red, the log2 of CNV ratio are arrounding 0.6~0.7.

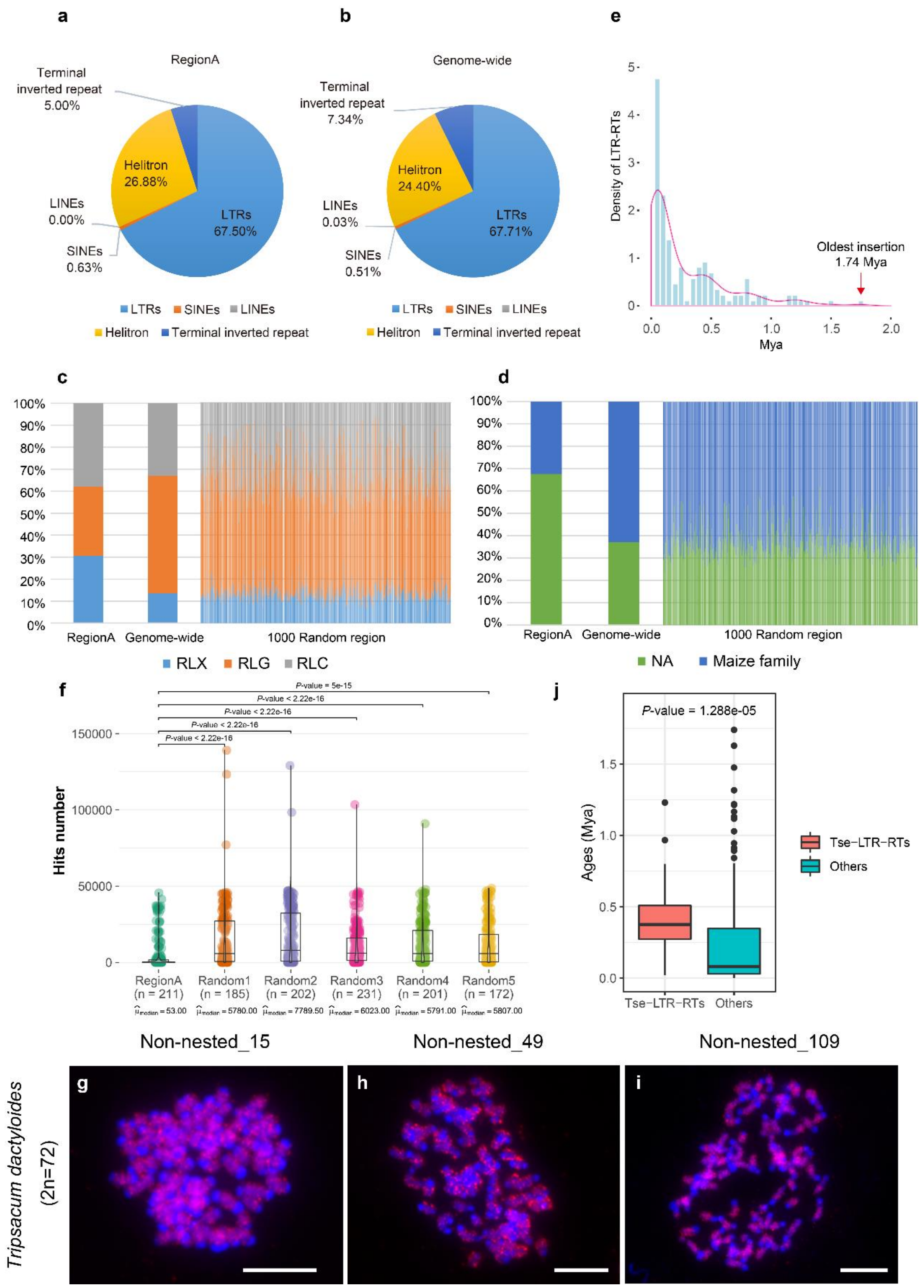

Figure S7. Characteristics of RegionA transposable elements. Percentages of types of (a) RegionA and (b) genome-wide transposable elements. (c) Comparison of LTR retrotransposons superfamilies in RegionA, genome-wide and 1000 random region. (d) Comparison of LTR retrotransposons families in RegionA, genome-wide and 1000 random region. (e) Distribution of insertion timing of LTR-RTs in RegionA. (f) Comparisons of genome wide hits number for LTR-RTs in RegionA and 5 random region. RegionA LTR-

RTs showed significantly lower hits number compared with other random region (Mann–Whitney U test, MWU, only significant comparisons were shown). (g-i) FISH analysis of 3 representative Tse-LTR-RTs cloned from RegionA to *Tripsacum dactyloides* (2n=72). All three LTR-RTs showed a genome-wide distribution pattern on T. *dactyloides* chromosomes. Scale bar = 10μm. (j) Ages comparison of Tripsacum-specific enriched LTR-RTs (Tse-LTR-RTs) with other LTR-RTs. The Tse-LTR-RTs were estimated with older insertion age than other LTR-RTs in RegionA (Mann–Whitney U test, MWU).

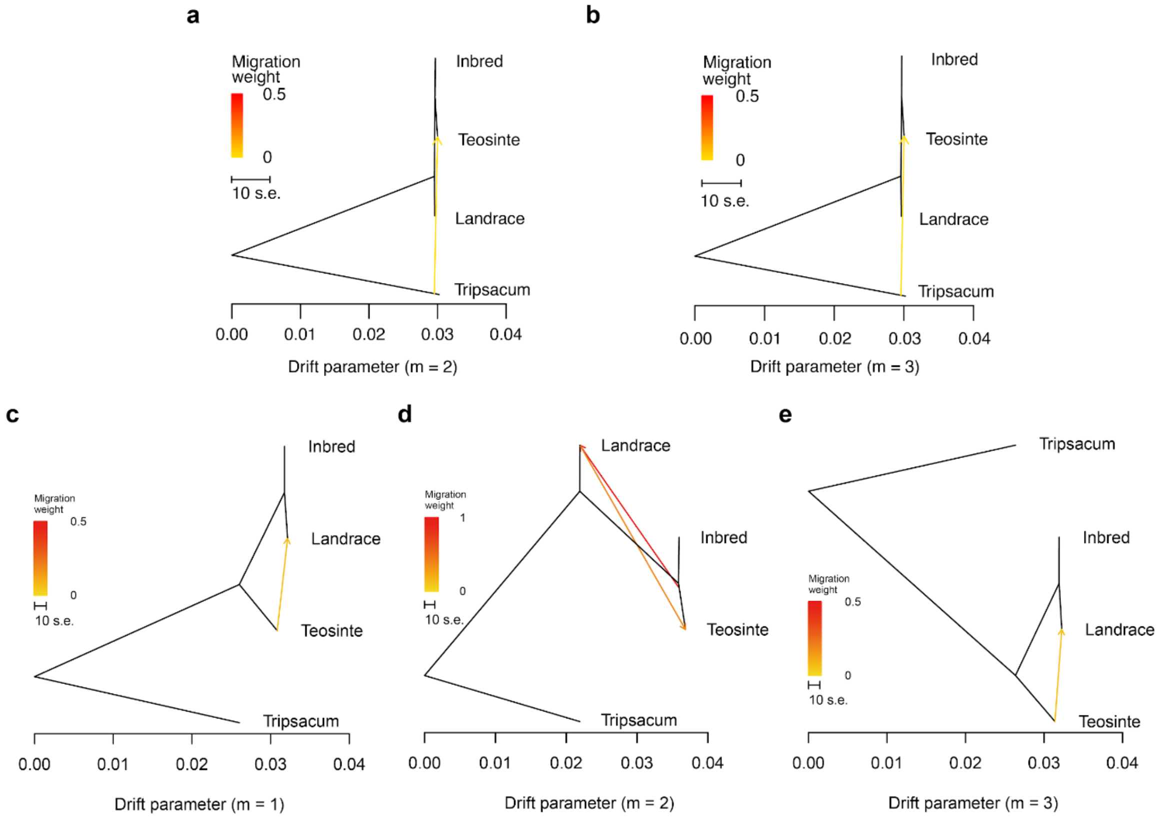

Figure S8. Gene flow detected among *Tripsacum* and *Zea* for (a-b) RegionA when (a) m=2 and (b) m=3; for (c-e) maize whole chromosome 6 when (c) m=1, (d) m=2 and (e) m=3. One to three migration events were set for analysis. Directions of gene flow were indicated by arrows.

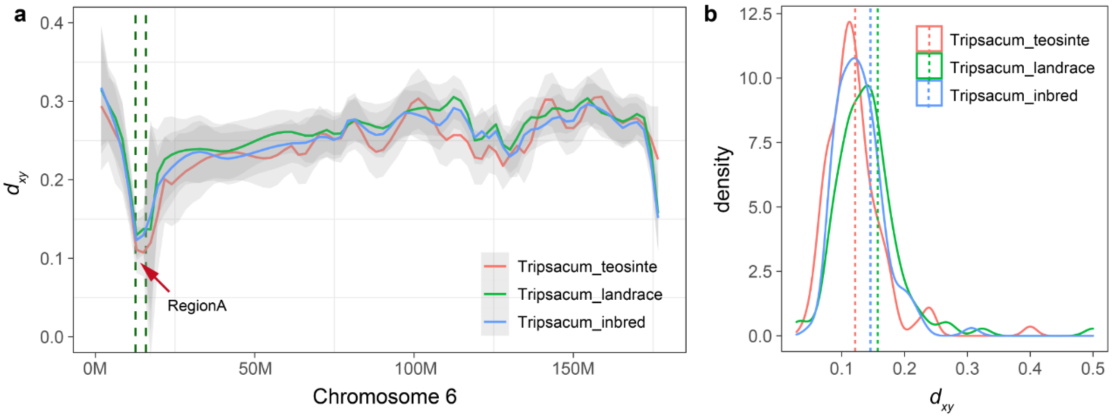

Figure S9. Absolute sequence divergence (d_{xy}) between *Tripsacum* and *Zea*. (a) d_{xy} values between *Tripsacum* and *Zea* across maize chromosome 6. RegionA is indicated by two vertical lines. (b) Density plot of d_{xy} value of RegionA windows. The dashed vertical lines indicated the cutoff of the whole genome bottom 5% d_{xy} value.

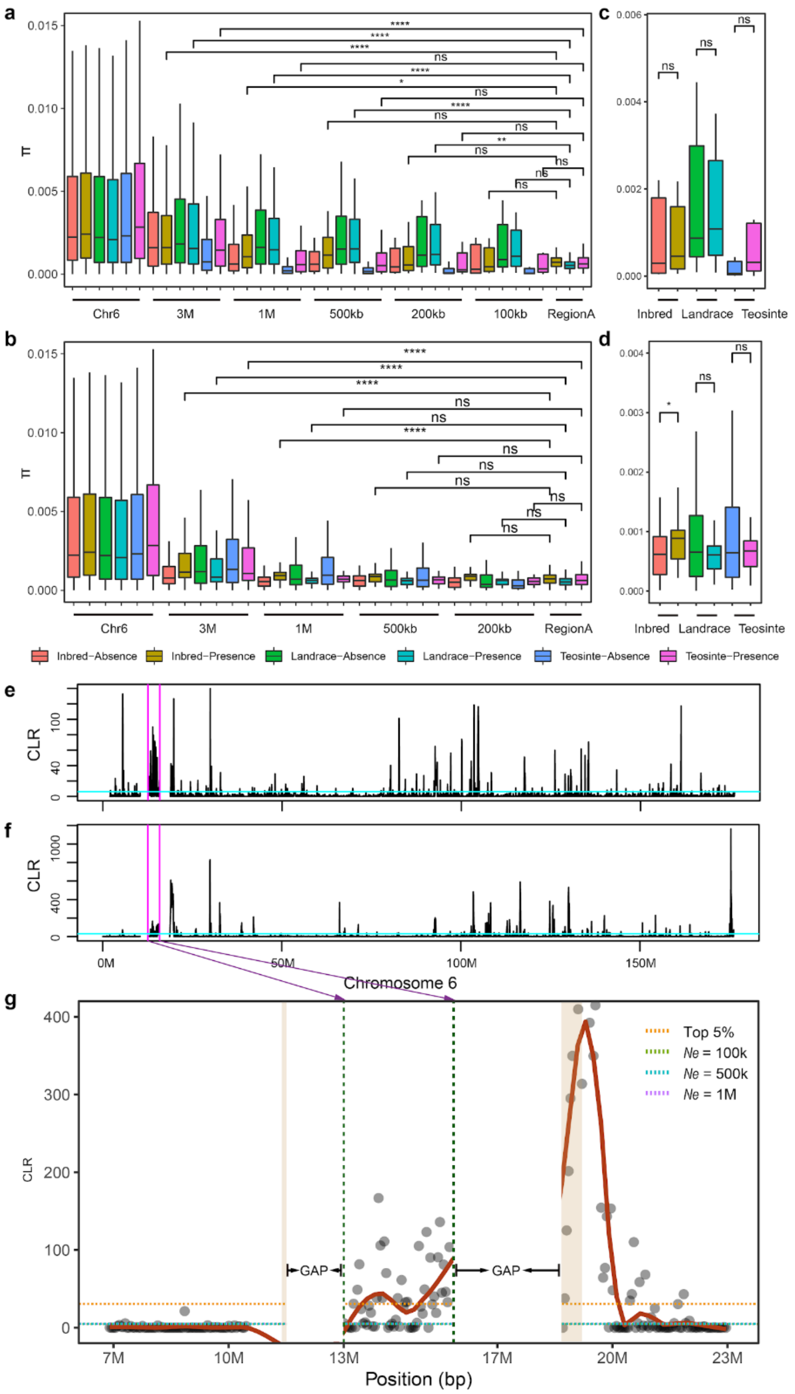

Figure S10. Genetic diversity and selection signals among different populations. (a-b) Pairwise comparison of nucleotide diversity between RegionA and upstream (a) or downstream (b) flanking regions with different scales. (c-d) Pairwise comparison of nucleotide diversity between presence-line and absence-line in 100kb upstream flanking region (c) or 500kb downstream flanking region (d). All comparisons were conducted with Wilcoxon rank sum test, ns: p > 0.05, *: p <= 0.05, **: p <= 0.01, ***: p <= 0.001, ****: p <= 0.0001. (e-f) Positive selection analysis of chromosome 6 in modern inbred (e) and landrace (f). Cyan line indicates the cut-off of the top 5% CLR value for whole genome, two pink vertical lines indicate RegionA. (g) A zoomed-in view of the region from 7Mb to 23Mb in landrace.

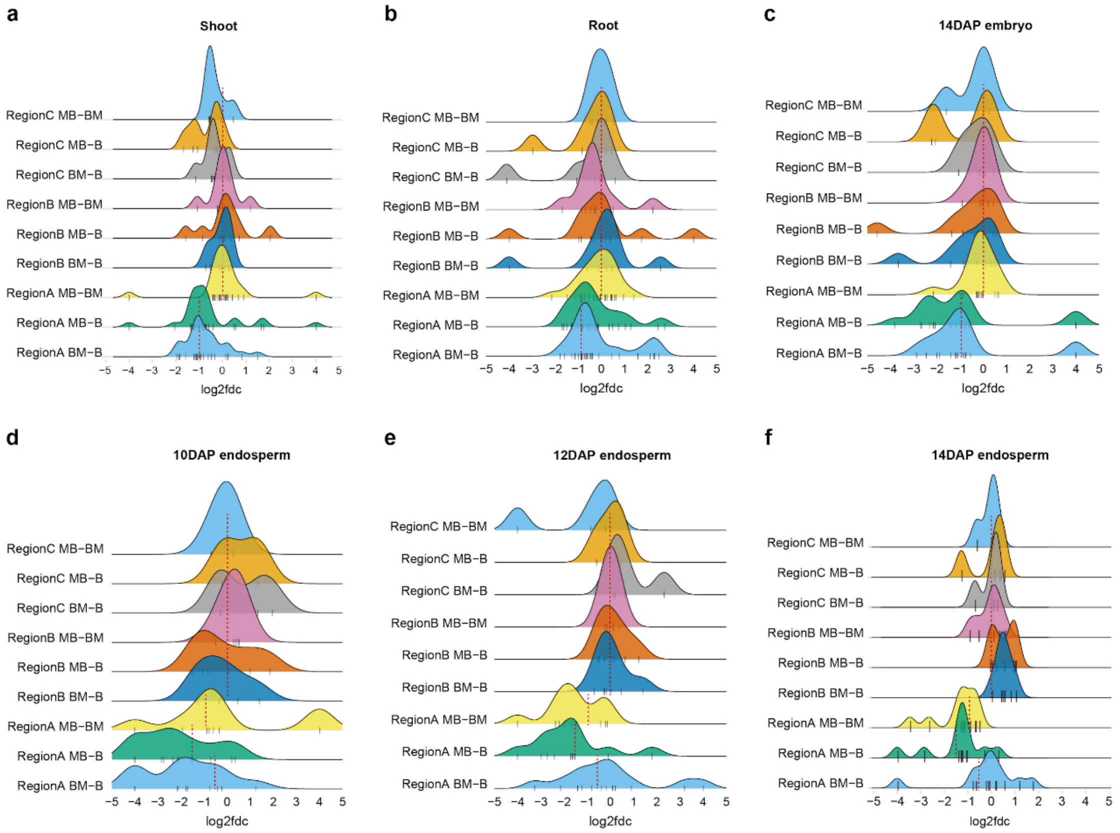

Figure S11. Transcription profiling of the genes in PAVs by RNA-seq for (a-c) diploid cells and (d-f) triploid cells. Gene expression was compared among B73 and reciprocal B73/Mo17 hybrids in shoot (a), root (b), 14 DAP embryo (c), 10 DAP endosperm (d), 12 DAP endosperm (e) and 14 DAP endosperm (f), and density of genes for different groups was shown. X-axis represented the log2 of fold change of genes for different groups of comparison, red dashed line represented the log2 of fold change value for standard dosage ratio in different types of tissues.

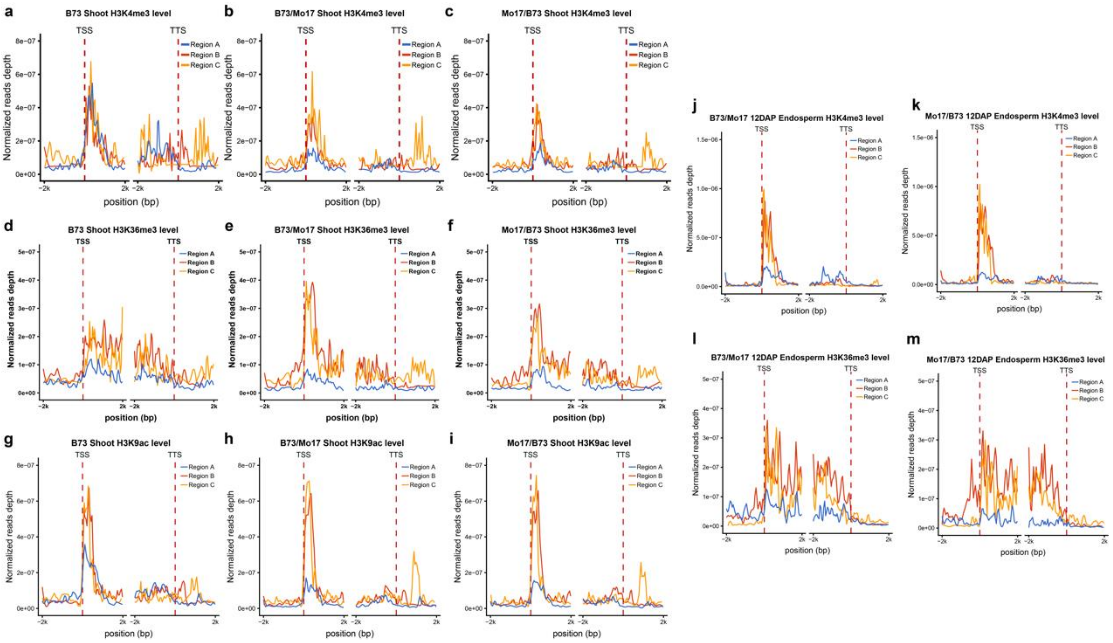

Figure S12. Histone modification change with PAVs genes in (a-i) seedling shoot (diploid cells) and (j-m) 12 DAP endosperm (triploid cells) dissected from B73, Mo17 and the reciprocal hybrids. (a-c) Shoot H3K4me3 modification; (d-f) shoot H3K36me3 modification; (g-i) shoot H3K9ac modification; (j-k) 12 DAP endosperm H3K4me3 modification; (l-m) 12 DAP endosperm H3K36me3 modification. The average modification of RegionA but not RegionB and RegionC showing a reduction that was correlated with the linear dosage of transcription activity.

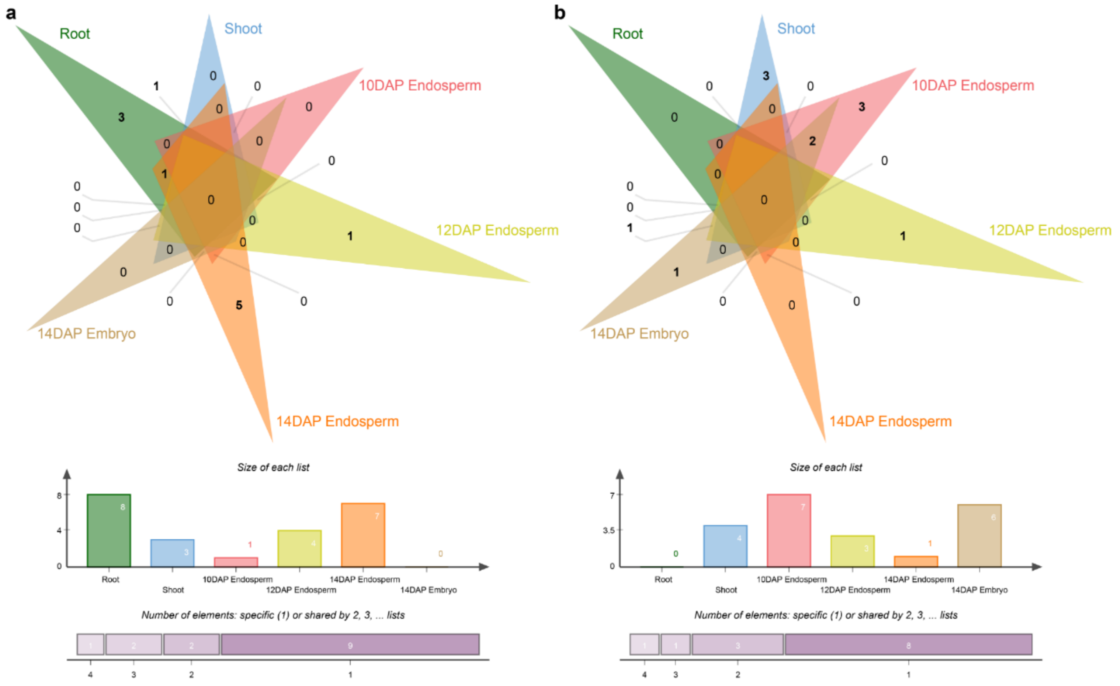

Figure S13. Venn diagram of intersections of non-additive RegionA genes in different tissues. (a) Non-additive (higher) genes; (b) Non-additive (lower) genes. These non-additive genes showed high tissue specificity.

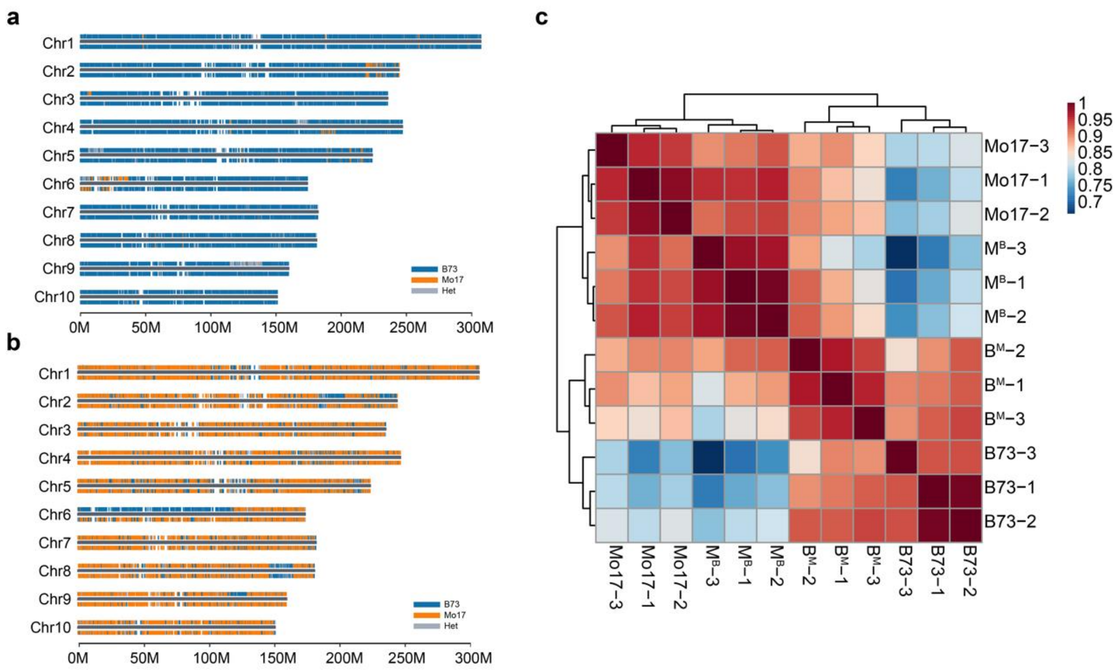

Figure S14. Schematic diagram shows genotype of the B73-Mo17 Near-Isogenic Lines (NILs) and their parental inbreds selected for transcriptome sequencing. For each chromosome, (a) above the gray horizontal line is B^M, while below is B73; (b) above the gray horizontal line is M^B, while below is Mo17. (c) Comparison of Pearson correlation patterns among different samples.

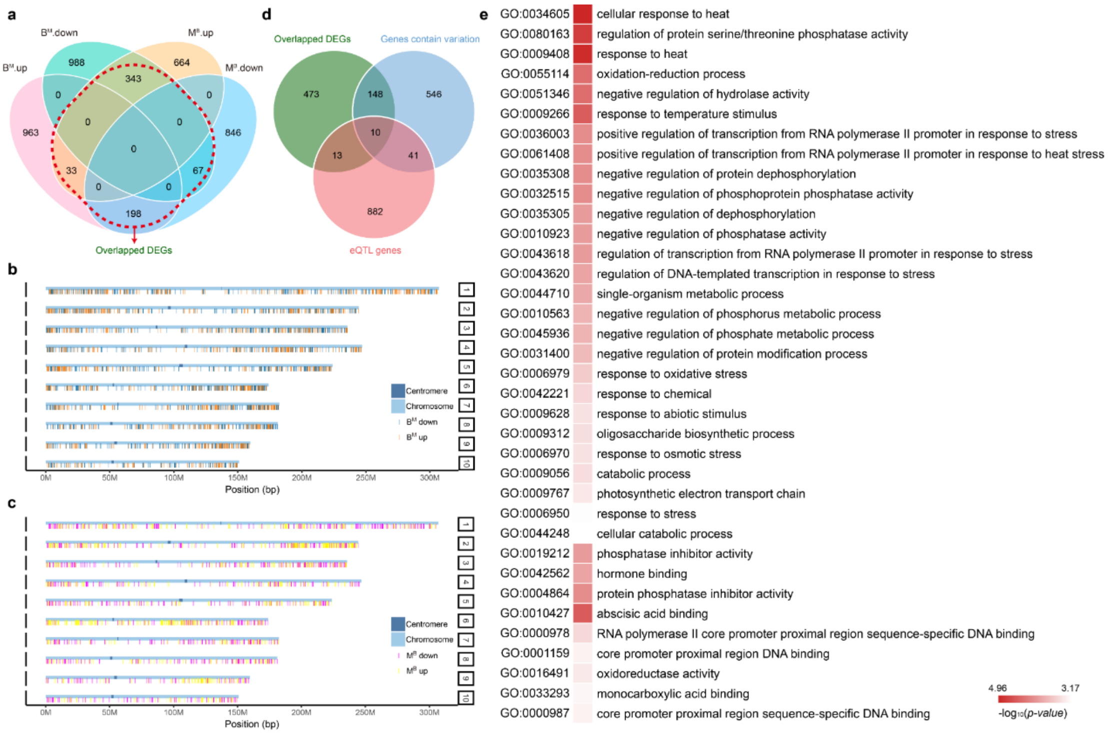

Figure S15. Transcriptomic response resulting from the RegionA PAV on the genome background. (a) Venn diagram shows intersections between datasets of differentially expressed genes (DEGs). DEGs were identified by transcriptomic comparison of B^M versus B73 inbred, and M^B versus Mo17 inbred, respectively. Red dashed box indicated overlapped DEGs. (b-c) Genome wide distribution of differentially expressed genes (DEGs). (b) DEGs of B^M, (c) DEGs of M^B. (d) Venn diagram of intersections of overlapped DEGs and genes with differential genetic background or eQTL genes, 473 genes stripped from that for downstream analysis. (e) Gene ontology (GO) analysis of eventual DEGs. The color of each cell indicates $-\log_{10}$ (P-values) of GO enrichment according to the scale shown.

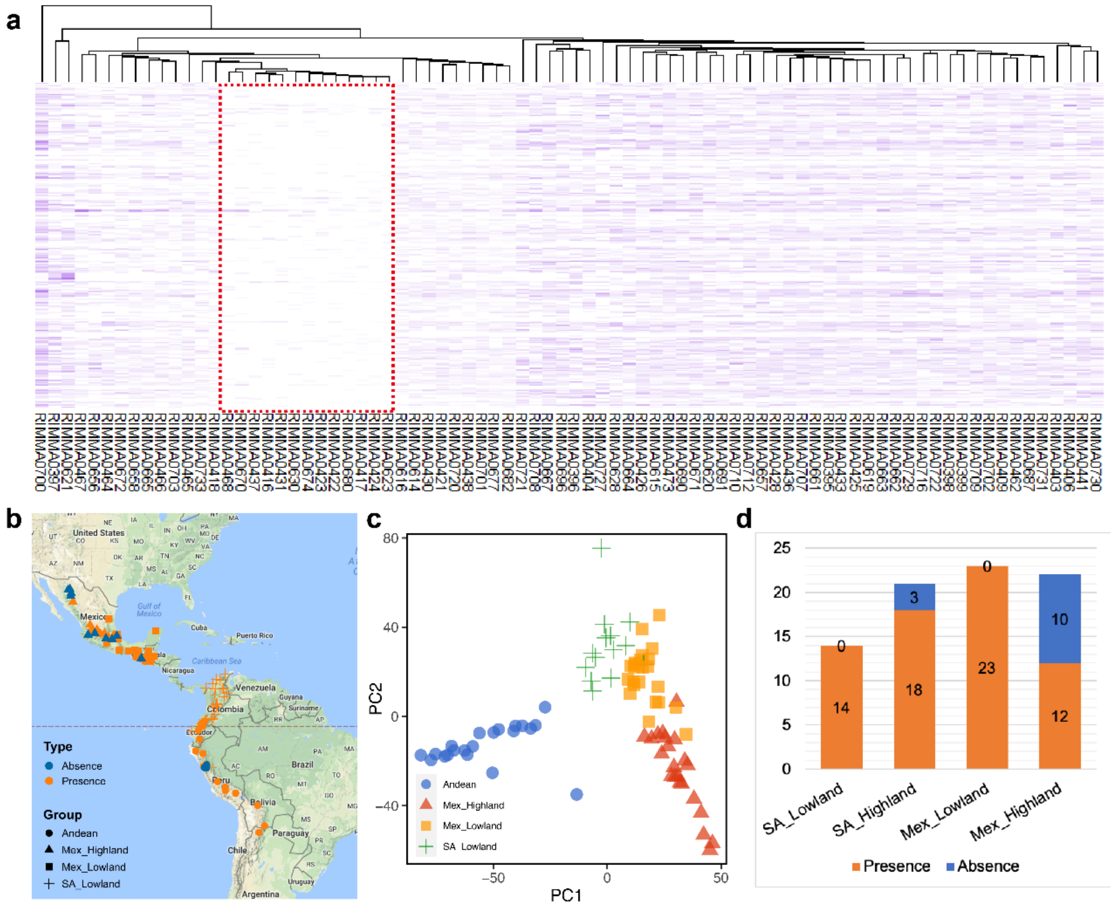

Figure S16. Presence or absence of RegionA was significantly associated with altitude of maize landrace. (a) Presence absence identification of RegionA among 80 maize landrace accessions. Each column represents one line, each row represents normalized read depth of one 10kb window in RegionA, all lines were clustered using R package *pheatmap* with hierarchical clustering method. Red dashed box indicated absence lines. (b) Geographic locations of 81 maize landrace accessions, consisting of four populations. Abbreviations: *Mex_Highland* : Mexican highlands (average altitude of samples : 2,147m), *Mex_Lowland* : Mexican lowlands (average altitude of samples : 557m), *SA_Lowland* : South American lowlands (average altitude of samples : 656m), *Andean* or *SA_Highland* : Andean highlands of South America (average altitude of samples : 2,504m). Blue dots represent absence lines, orange dots represent presence lines. (c) PCA plots of maize landrace. Different populations are represented in different colors and shapes as shown. (d) Comparison of the number of presence lines and absence lines at different geographic areas. RegionA is only absent from the highland landraces.

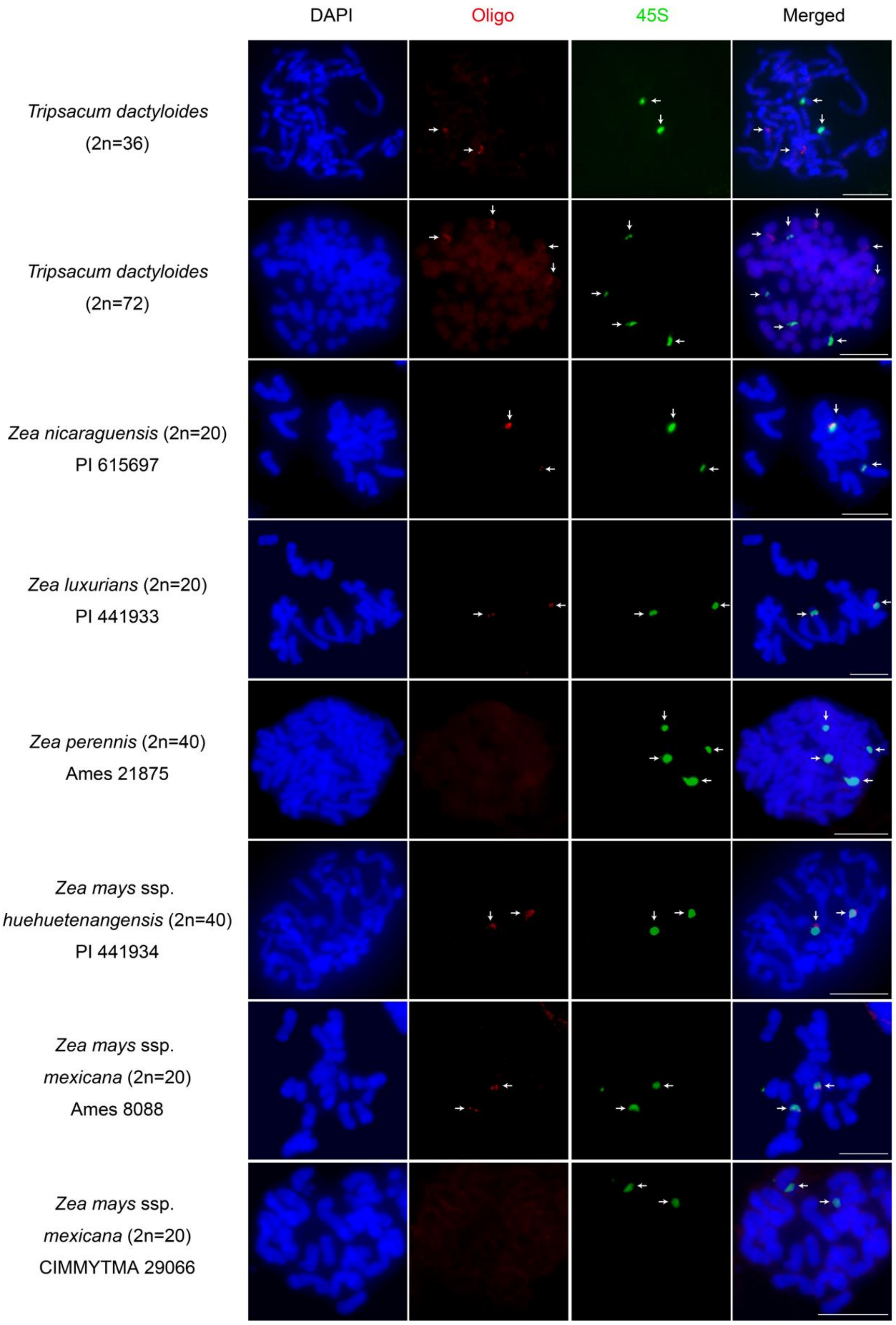

Figure S17. FISH analysis of the relative localization of the RegionA (red signal, marked as Oligo) and 45S rDNA (green signal, marked as 45S) on metaphase chromosomes of *Zea* genus and *Tripsacum*. DAPI, RegionA (red) and 45S rDNA(green) fluorescence signals were digitally separated from Merged. White arrows indicate the RegionA and 45S signals we detected. Signals of RegionA and 45S rDNA were juxtaposed on *Zea* genus chromosomes. Scale bar = 10μm.
