# Supplementary material for: Megabase-scale presence-absence variation with Tripsacum origin was under selection during maize domestication and adaptation
Source: Genome Biol. 2021 Aug 20;22:237. doi: 10.1186/s13059-021-02448-2 (PMC8377971; doi:10.1186/s13059-021-02448-2)
Supplement: Supplementary file 3 — Additional file 3. Review history. [file 13059_2021_2448_MOESM3_ESM.docx]

Review History

**First round of review**

**Reviewer 1**

**Are you able to assess all statistics in the manuscript, including the appropriateness of statistical tests used?**

**There are no statistics in the manuscript.**

**Comments to author:**

In this paper, Huang et al. identify putative and large (> 500 kb) presence-absence variants (PAVs) in maize. They begin by contrasting B73 to Mo17 to identify putative regions. They then focus on one particular PAV (region A) by mapping to a broader sample of individuals, including landraces and inbred lines, and trying try to decipher its origin, content, recombination history and potential function.

There are some interesting observations in this paper, particularly (in my view) that the region seems to be enriched for Tripsacum-like transposable elements. I thus believe it is a region that deserves attention and focus. However, I was not convinced by some of the conclusions in the manuscript, ranging from the odd pattern of coverage in putative heterozygotes, to claims about selection, repressed recombination and introgression.

1. In my view, the emphasis in this paper on presence-absence variation is a little bit misguided, seeing that the original identification of these variants take place in an artificial system (inbred lines). I don't doubt that real PAVs have been found, given the starting materials. However, as a consequence of the starting material there is not a good idea whether these are PAVs in real diploids or simply hemizygous regions (although see comments on Figure 1 below). This is (in some ways) simply semantics, but in other ways the major justification for the paper is that large PAVs are particularly interesting. It might be better to focus on large structural variants (SVs) more generally?

2. There has been lots of recent work on structural variants (SVs) that go beyond pan-genomes and into the evolution of such regions. Unfortunately, some important literature was not cited. I'd particularly suggest work by Zhou et al on grapes (in Nature Plants) and Kou et al rice (in MBE), which document PAVs and SVs with long and short read data. Some of the wonderful work on maize SVs (particularly a large inversion) and environmental adaptation from the Ross-Ibarra lab also were not mentioned, I believe. There is also a long historical literature on the malleability of maize genomes, which seems to be pertinent here - e.g., papers (again) from the Ross-Ibarra lab, Roessler et al. 2019 Nature Plants and citations therein. These clearly indicate that gain and loss of genomic regions are very common, supporting the basic observation of this paper but also probably strongly suggesting that starting with the B73 / Mo17 comparison is likely to greatly underestimate the kind of phenomenon the authors are interested in. More generally, I felt as comments like (l. 77 and elsewhere throughout the ms) "the rarity of large size PAVs in plants" are perhaps misleading, given that papers like this one are likely to have begun with genetic material that misrepresent the extent of these phenomena.

3. The five regions mentioned in lines 119 to 123 are interesting. This section needs some brief explanation of how they were found (whole genome alignment or what?), so that the reader can follow how the inferences were made. I really like lines 142 to 154…

4. Figure 1d. Since no lines (even heterozygous landraces) have coverage around 50%, I believe this implies that region A is never heterozygous. If true, this implies some sort of meiotic drive mechanism and/or heterozygote lethality. But then again, BM hybrids (line 340) have intermediate expression. So, I'm perplexed on a few levels, with apologies if I am simply misunderstanding. My questions are: Is there evidence for heterozygote lethality in literature for this or any other regions? This should be mentioned or discussed, and the fundamental observation (no heterozygotes) should be discussed, too. Second, I was a bit concerned that this result (the lack of 50% coverage in real diploids) was an artifact. I have no evidence toward that end, of course, but the observation is so striking that it requires some justification. Third, I was surprised that only one Tripsacum line was shown. I believe there were more in the Supp figure. All should be shown here, I think, but it is not a big deal. The nice thing about this graph, though, it that it suggests that the region A analysis was not just an artifact of the inbreeding process that created B73 and Mo17 (inbreeding may select for or initiate SV events), but it is instead more general.

5. Line 176 - are the NAM founders based on long-read data? Some details here would be helpful.

6. Line 189 - what does major mean in this context? Can you give a range or percentage?

7. Line 229 - sorry if I am misunderstanding, but I think the argument is that region A is enriched for Tse-LTRs. If so, why weren't there clear FISH signals in region A? Nonetheless, the blast results based on Tsp-LTRs (lines 232 to 233) do suggest a strange history for this region.

8. Figure 2a - I don't understand how tripsacum specific LTRs can be found in maize. Aren't they then just Tripsacum enriched? The overlap between Tsp and Zea LTRs seems to disagree with the definition of Tsp on line 225.

9. I do not believe figure 2h constitutes strong evidence of introgression. One might try ABBA-BABA tests. I also think that measuring sequence divergence in this region (compared to the rest of the genome) could help suggest that introgression occurred. Overall, the paper (rightly) concludes in the Discussion that one cannot really differentiate between introgression and shared ancestor, based on the evidence presented. For that reason, I think that lines 250 to 254 are overstatements.

10. With apologies, I did not understand what I, as a reader, was supposed to glean from the coverage information in Figure 2i. Is it to imply that there are small SVs within this larger region? Why are there so many genes in B73 that are not present in Tripsacum. Is it just annotation differences?

11. The recognition that RegionA is imbedded in an NOR and part of the 45S rDNA array is quite interesting. Figure 3a suggests that RegionA is fairly typical, in terms of low recombination rate regions in the genome.

12. I do not believe the selection analyses in lines 287 to 330. By definition, a presence variant is going to have a lower pi, because it has a lower population size. The slightly compelling observation from this analysis is that the frequency of the presence variant increases from teosinte to landraces, but this is not uncommon for many variants (due to genetic drift and demographic effects). I didn't understand the significance of the note in lines 311 to 313; is this necessary or can it be explained better? In short, to make this inference properly requires more sophisticated methods than reports of pi and D; simulations might be one approach, but they will require much more careful exposition of null hypotheses.

13. Line 344-347. I don't understand the significance of looking at heterochromatic modifiers in this context, since they aren't discussed more carefully. It is not that it is in any way incorrect to report the data, but they don't contribute to the story.

14. Line 348 - I presumed nonlinear outliers are non-additive (or mid-parent) values? This could be worded more clearly.

15. In my view, lines 353 to 355 doesn't implicate Region A in any particularly way. Is region A particularly prone - relative to the genome pattern - to have such outliers?

16. I am concerned about the analysis of NILs. If I understand correctly, the filtering mechanism cannot account for trans-acting effects in imperfect backgrounds. (This is Mo17 alleles in the non-A region of the B_m line that drive observations of differential expression.) As such, I think concluding that the A region plays a role in the 486 DEGs is possibly exaggerated and perhaps wrong.

17. Line 402. One of the things about PAVs - and their apparent rarity - is not only one of semantics (see point #1 above) but the lack of good data. I wonder if this point, which is made nicely in lines 430 to 432, could be moved here?

18. I like the equanimity of lines 456 to 460. Indeed, while I agree with both models, I find the evidence for introgression to be tentative at best.

**Reviewer 2**

**Are you able to assess all statistics in the manuscript, including the appropriateness of statistical tests used?**

Yes, and I have assessed the statistics in my report.

**Comments to author:**

In this manuscript, Huang et al. updated the maize B73 reference genome using Hi-C data and conducted genomic analyses on big SVs. The authors found some interesting SV patterns in domestication of maize and zea. I think the manuscript was well written. However, many parts of the evolutionary genetic analyses were unprofessional and conclusions draw form it was misleading and incorrect. I also have other major and minor comments for the authors to consider.

Major:

1. The authors seem don't familiar with the literature on structural variation and on zea evolution. Some fundamental papers on SV and plant evolution and on zea evolution were not referred in this paper, so that the authors used "novel", "unknown" and similar words across the paper. I recommend the authors to rewrite the manuscript, especially the introduction and discussion part, based on a better understanding on the recent process in the field.

2. The evolutionary genomic analyses were unprofessional, and the conclusions draw form it was misleading and incorrect at many places. For example, the authors calculated the population mutation rate and genetic diversity at the big SV region and argued that the SV regions were low in diversity and under selection. A given SV is a single locus (like a SNP) with all sites highly linked within it. So, it has nothing to do with selection, it is just a common feature of SV. Another example the zea migration part. I don't think the authors had enough samples or analyses to reach this conclusion.

Minor:

L19: What is "environmental plasticity"

L27: What does "evolution erosion" mean? Please write clearly using filed-specific words

L29: Introgression from distant-related species is a very common feature in plants, what is "unknown"?

L36: The "fossil" SV?

L41: Paper 1,2 have nothing to do with SVs

L42-43: This is incorrect. Please read recent review papers on plant SVs carefully.

L53-54: This is incorrect too. Please read recent studies in rice, maize, tomato and grape.

L55-62: The citations are missing in this paragraph and many of the arguments are wrong.

L63-781: The whole introduction is on PAV not SV, but the paper was titled with SV. Please note that PAV ≠SV.

L79 and following in the same paragraph: Please cite the original papers in each sentence.

L115: This had been done several times already in previous publications, why again?

L116-182: Why region A-E were chosen, there was very strong bias that comparing Mo17 and B73 and used the PAV markers in the multi-species diversity panel.

L182-L254: The analyses done here was not enough to support the authors arguments.

L286-330: Please see my major comment 2

L 331-355: This is a common feature of PAV.

L356: What is "environmental fitness", do the authors mean environmental adaptation?

L433: See my major comment 2

L526: There is a non-gap assembly of B73 based on deep Pacbio sequencing and scaffolding (V5). Why the authors chose to update an old version?

Fig. 6 is misleading!

**Authors Response**

**Point-by-point responses to the reviewers’ comments:**

Reviewer #1:

In this paper, Huang et al. identify putative and large (> 500 kb) presence-absence variants (PAVs) in maize. They begin by contrasting B73 to Mo17 to identify putative regions. They then focus on one particular PAV (region A) by mapping to a broader sample of individuals, including landraces and inbred lines, and trying to decipher its origin, content, recombination history and potential function. There are some interesting observations in this paper, particularly (in my view) that the region seems to be enriched for Tripsacum-like transposable elements. I thus believe it is a region that deserves attention and focus. However, I was not convinced by some of the conclusions in the manuscript, ranging from the odd pattern of coverage in putative heterozygotes, to claims about selection, repressed recombination and introgression.

1. In my view, the emphasis in this paper on presence-absence variation is a little bit misguided, seeing that the original identification of these variants take place in an artificial system (inbred lines). I don't doubt that real PAVs have been found, given the starting materials. However, as a consequence of the starting material there is not a good idea whether these are PAVs in real diploids or simply hemizygous regions (although see comments on Figure 1 below). This is (in some ways) simply semantics, but in other ways the major justification for the paper is that large PAVs are particularly interesting. It might be better to focus on large structural variants (SVs) more generally?

*Reply: Thanks a lot for the reviewer’s comments. We chose the starting materials, inbred B73 and Mo17, which represent the two best-known maize variety groups and are the most common pair of maize inbred lines in many genetic and molecular studies. Moreover, their high-quality de novo assembled genomes are available to ensure the accuracy of our research. Starting with these large-size PAVs identified from two inbreds, we further characterized the presence or absence in a diversity panel. Particularly for RegionA, the NGS data coverage, the syntenic comparison between high-quality maize genome and FISH, all these data confirmed the high integrity for this fragment, which presented as either nearly complete presence or absence in the natural diversity panel. As the Reviewer suggested, we also characterized the genotype of RegionA for the HapMap2 lines and others with available sequencing resources, and indeed found heterozygous in a small group of Meso- and South America lines (see Reply 4).*

2. There has been lots of recent work on structural variants (SVs) that go beyond pangenomes and into the evolution of such regions. Unfortunately, some important literature was not cited. I'd particularly suggest work by Zhou et al on grapes (in Nature Plants) and Kou et al rice (in MBE), which document PAVs and SVs with long and short read data. Some of the wonderful work on maize SVs (particularly a large inversion) and environmental adaptation from the Ross-Ibarra lab also were not mentioned, I believe. There is also a long historical literature on the malleability of maize genomes, which seems to be pertinent here - e.g., papers (again) from the Ross-Ibarra lab, Roessler et al. 2019 Nature Plants and citations therein. These clearly indicate that gain and loss of genomic regions are very common, supporting the basic observation of this paper but also probably strongly suggesting that starting with the B73 / Mo17 comparison is likely to greatly underestimate the kind of phenomenon the authors are interested in. More generally, I felt as comments like (l. 77 and elsewhere throughout the ms) "the rarity of large size PAVs in plants" are perhaps misleading, given that papers like this one are likely to have begun with genetic material that misrepresent the extent of these phenomena.

*Reply: Thank the reviewer for the constructive comments and the suggested references, which are now added in the revised manuscript, including but not limited to: [1,2] for population genomics of SVs; [3,4] for maize SVs; [5,6] for maize environmental adaptation; [7,8] for the malleability of maize genomes. We apologize for the misleading description of the "The rarity of large size PAVs in plants". What we meant to highlight is that such a large-scale gene-riched PAV shows high sequence integrity in the natural diversity panel is not common. In fact, RegionA, the largest PAV characterized between B73 and Mo17, is also the largest PAV found in maize population so far.*

*With the development of sequencing technology, a growing number of research about plant pan-genomic SVs across large population samples have been reported in recent years. However, these reports are focusing on the pattern of genome-wide spectrum or distribution in the level of population genetics, the particular focus on representative large-size PAVs and their biological insight has been rarely explored. In this report, we conducted an interdisciplinary study using cytogenetics, DNA sequencing, genome assembly, population genetics, phylogenetics, and evolutionary comparisons to comprehensively focus on RegionA particularly, which showed unparalleled features including the high sequence integrity across diversity populations and striking genomic footprints shared with Tripsacum. Moreover, for the first time we found its embedded location within the nucleolus organizer region (NOR), which was associated with the suppressed recombination and preserved the sequence footprint against the otherwise inevitable evolution variation. We also provide evidence about its role in environmental adaptation and agronomic traits. All the above insight illustrated by this particular large size PAV, instantiating a neglected view by the published genome-wide PAV studies.*

3. The five regions mentioned in lines 119 to 123 are interesting. This section needs some brief explanation of how they were found (whole genome alignment or what?), so that the reader can follow how the inferences were made. I really like lines 142 to 154…

*Reply: Thank you for your valuable comment. We added the details in the revised manuscript (Line122-126). For short, we used a sliding-window method that was previously mentioned to identify B73 presence-absence variation [9], the B73 reference genome was divided into 500bp windows with a step size of 100bp and aligned to B73 and Mo17 genome respectively with Bwa mem. The windows that could not be aligned to the Mo17 genome but could be properly aligned to the B73 genome were defined as B73-specific sequences, the specific sequences within 100 kb of the physical coordinates were merged, the merged region that contains more than 10% PAV sequences was defined as a PAV cluster. Vice versa, the Mo17 presence-variation was identified through the same method.*

4. Figure 1d. Since no lines (even heterozygous landraces) have coverage around 50%, I believe this implies that region A is never heterozygous. If true, this implies some sort of meiotic drive mechanism and/or heterozygote lethality. But then again, BM hybrids (line 340) have intermediate expression. So, I'm perplexed on a few levels, with apologies if I am simply misunderstanding. My questions are: Is there evidence for heterozygote lethality in literature for this or any other regions? This should be mentioned or discussed, and the fundamental observation (no heterozygotes) should be discussed, too.

*Reply: Thank you for your valuable comment. In fact, the coverage (proportions of presencebin in RegionA) was just used to report the percentage of RegionA sequences exist in different lines, but it can’t distinguish homozygous from heterozygous, therefore it may not be used as an indicator of heterozygosity. To address whether there are RegionA heterozygotes in maize landrace, we conducted copy number variation analyses by cnv-seq [10] using the maize natural variation population (100 HapMap2 line and 99 Meso- and South America landrace) as treats, B73 as a reference. The results showed the mean log2 value of CNV ratio for the whole genome are around 0 for each line, which is consistent with the maize ploidy as expected. But for RegionA, according to the results of k-means clustering, it can be easily distinguished into three genotypes: Homozygous absence (0/0), the log2 of CNV ratio are around the range of -2 ~ -4; Homozygous presence (1/1), the log2 of CNV ratio are around the range of 0.6 ~ 0.7; Heterozygous (0/1), the log2 of CNV ratio are approximal -0.3, which showed about half of CNV ratio relative to RegionA-presence homozygous (Supplementary Fig. 6b). The log2 of CNV ratio of Homozygous presence (1/1) genotypes were slightly higher than the whole genome (0.6 verus 0), possibly due to the extremely low genetic diversity of RegionA, so that RegionA contained more reads with high-quality alignments than the rest of the genome. Taken together, the above results showed that no RegionA heterozygotes were found in 100 HapMap2 lines, but 26 out of 99 Meso- and South America lines were defined as RegionA heterozygotes. Given that RegionA heterozygotes landrace lines existed, in addition to the fact that RegionA presence inbreds can cross successfully with absence inbreds as fertile hybrid (like B73×Mo17), we rule out the possibility of heterozygote lethality. We added these results to the main text (Line 204 - 207): “...We also identified the RegionA genotypes in the diversity panel of maize, the results showed that no RegionA heterozygotes were found in 100 HapMap2 lines, but 26 of 99 Meso- and South America lines were defined as RegionA heterozygotes (Supplementary Fig. 6)….” Supplementary Figure 6. RegionA genotypes in the diversity panel of maize. (a) Dot plot of log2(CNV ratio) for each line. The y axis is the log2(Treat/B73 read numbers), x axis is the scale of chromosome 6 from 10M to 18M, RegionA is indicated by two green vertical dash lines, each dot represents a 100-kb window, red line indicates the fitting curve for RegionA windows. (b) Density plot of CNV ratio for RegionA windows. Each curve represents a line, vertical dash lines represent median value for each subgroup.*

Second, I was a bit concerned that this result (the lack of 50% coverage in real diploids) was an artifact. I have no evidence toward that end, of course, but the observation is so striking that it requires some justification.

*Reply: To justify the striking results that no lines were found to contain an intermediate proportion for presence-bin, we made the clarification through the following aspects: 1) We increased the sequence coverage for our HapMap2 lines with HapMap3 data and re-analysis the PAV region (Fig 1d, Supplementary Table. 1); 2) The identification of PAV genes in RegionA based on CDS coverage also showed consistency with our findings: the majority of RegionA genes are completely present or absent in diversity lines (Supplementary Fig. 5a); 3) The syntenic comparison between B73 and 25 highquality maize NAM founders genome (de novo assembled using PacBio long reads a mate-pair strategy) [11] also confirms the integrity of the RegionA (Supplementary Fig. 4); 4) We also performed FISH using RegionA-specific oligo probes for some maize inbred lines for cytogenetic validation, the results were consistent with our bioinformatics analysis (Supplementary Fig. 5b-e). We added these data to the main text of the revised manuscript. Please see Line187- 193 read as “...We further investigated the coding sequence coverage of RegionA genes by mapping HapMap2 resequencing reads to B73 reference genome, the results showed that differed from RegionB and RegionC, the majority of RegionA genes are nearly completely present or absent in diversity lines (Supplementary Fig. 5a). In accordance with that, FISH experiment using RegionA-specific oligo probes showed signals in the RegisonA-present inbreds while none detected in the absent lines (Supplementary Fig. 5b-e)....”*

*Supplementary Figure 5. The majority of RegionA genes are either completely present or absent in diversity lines. (a) Coverage distribution of genes within 3 largest B73 PAV in HapMap2 lines. For each gene, the coverage of CDS region was shown, columns in the heatmap show HapMap2 lines and the rows represent genes located in these PAVs. (b-e) FISH validation using RegionA-specific oligo probes in selected maize inbred lines. RegionA-specific signals (red) were observed on a pair of homologous chromosomes of B97 and Chang72 mitosis metaphase cells (b and c), while absent on P39 and OH43 metaphase chromosomes (d and e). Scale bars = 10μm.*

Third, I was surprised that only one Tripsacum line was shown. I believe there were more in the Supp figure. All should be shown here, I think, but it is not a big deal. The nice thing about this graph, though, it that it suggests that the region A analysis was not just an artifact of the inbreeding process that created B73 and Mo17 (inbreeding may select for or initiate SV events), but it is instead more general.

*Reply: Thank you for your kind suggestions, we have made changes accordingly by adding more Tripsacum lines as suggested (Fig 1d).*

5. Line 176 - are the NAM founders based on long-read data? Some details here would be helpful.

*Reply: Yes. The NAM founder genome project sequenced and assembled the 25 NAM founder inbreds using PacBio long reads by a mate-pair strategy, the resource is available at [11], and the genomes assembly are all high-quality with high completeness. These details have been included in the revised manuscript (Line 183).*

6. Line 189 - what does major mean in this context? Can you give a range or percentage?

*Reply: Thank you for your comments. We clarified this point by adding info to the new sentence reads as “..Interestingly, 4 of 4 (100%) Tripsacum and the one Zea luxurians contained major portions (76%~80%) of RegionA.”*

7. Line 229 - sorry if I am misunderstanding, but I think the argument is that region A is enriched for Tse-LTRs. If so, why weren't there clear FISH signals in region A? Nonetheless, the blast results based on Tsp-LTRs (lines 232 to 233) do suggest a strange history for this region.

*Reply: We apologize for the confusion. A description should have been included to note the technical limit by the FISH sensitivity. Actually, the minimum target DNA unambiguously detectable by FISH in plant mitotic metaphase chromosomes is about 4kb, and also it requires the targets are densely clustered [12]. However, the total number (216) of LTR-RTs in RegionA is low, and they are located separately. Moreover, we chose three single distinct Tse-LTRs as FISH probes respectively. For the single Tse-LTR, there are even fewer LTRs that belong to the same family may show high sequence identity as the FISH target. Due to the above technical limits, we were not able to detect unambiguous signals for any particular kinds of Tse-LTRs in RegionA by FISH.*

8. Figure 2a - I don't understand how tripsacum specific LTRs can be found in maize. Aren't they then just Tripsacum enriched? The overlap between Tsp and Zea LTRs seems to disagree with the definition of Tsp on line 225.

*Reply: We apologize for the confusion and misleading caused by ambiguously designated term as “tripsacum specific LTRs”. Our aim of this analysis was to identify the Tripsacum-presence LTRs which existed in Zea mays but not in other Zea species (in other words, low similarity), since these LTRs may represent the divergent expansion direction in the processing of Zea genus speciation. So we choose Zea luxurians as a control, and identified Tripsacum-specific presence LTRs (Tsp-LTRs) with the coverage of Tripsacum reads > 0.8 and the coverage of Zea luxurians reads < 0.8. In another word, these Tsp-LTRs can be found in Tripsacum and Zea mays, but not in the sister species other than Zea mays. Considering this piece of data doesn’t contribute much to the story, we removed the part of Tsp-LTRs and only kept Tse-LTRs in this version of manuscript to avoid misleading.*

9. I do not believe figure 2h constitutes strong evidence of introgression. One might try ABBA-BABA tests. I also think that measuring sequence divergence in this region (compared to the rest of the genome) could help suggest that introgression occurred. Overall, the paper (rightly) concludes in the Discussion that one cannot really differentiate between introgression and shared ancestor, based on the evidence presented. For that reason, I think that lines 250 to 254 are overstatements.

*Reply: Thank you for your comments. We agree that in addition to the Treemix results, further analysis should be deployed to clarify the gene migration from Tripsacum to maize. We actually have tried performing ABBA-BABA tests [13], but RegionA was not observed in the sorghum lines or other distantly related species (such as setaria or rice), so we can not find an appropriate outgroup as ancestral state control relative to Tripsacum. Alternatively we measured absolute sequence divergence (dxy) between Tripsacum and Zea in RegionA and compared it to the whole genome as you suggested. Obviously we can see that RegionA is a trough on the entire chromosome, and the dxy value of 60.5%~72.2% RegionA windows were below the cutoff of whole genome bottom 5% (Supplementary Figure 9). Since the reduced dxy has been generally considered as the sign of recent gene flow distinguished from shared ancestral variation [14,15], we attribute the dramatically reduced dxy between Tripsacum and Zea at RegionA to the recent coalescence at this region led by introgression. We agree that based on the current data, we still cannot determine which of the two scenarios is the real case in evolutionary history, but it seems more evidence favour the introgression scenario, as we proposed in the Discussion. We rephrased this point in the revised manuscript (Line 266-275, Line 474-478).*

*Supplementary Figure 9. Absolute sequence divergence (dxy) between Tripsacum and Zea. (a) dxy values between Tripsacum and Zea across maize chromosome 6. RegionA is indicated by two vertical lines. (b) Density plot of dxy value of RegionA windows. The dashed vertical lines indicated the cutoff of the whole genome bottom 5% dxy value.*

10. With apologies, I did not understand what I, as a reader, was supposed to glean from the coverage information in Figure 2i. Is it to imply that there are small SVs within this larger region? Why are there so many genes in B73 that are not present in Tripsacum. Is it just annotation differences?

*Reply: We totally understand the reviewer’s concern. The coverage information indicates the extremely high sequence similarity between Tripscaum and Zea although there are some small differences. These differences may be due to the genomic sequences variations between the modern Tripsacum line we used to sequence and the Tripsacum ancestor. Moreover, RegionA is not protected by rDNA in Tripsacum, which can make even more structural variations between it and modern maize inbred (B73). To date, no reference genome of Tripsacum has been completed yet, we only had to align the Tripsacum short-read data to B73 genome, so some parts of the region may not be covered. Due to the same reasons, the gene annotation for Tripsacum may not be complete or accurate, leading to the pattern shown in the Fig2i that a few putative homolog genes are missing in Tripsacum.*

11. The recognition that RegionA is imbedded in an NOR and part of the 45S rDNA array is quite interesting. Figure 3a suggests that RegionA is fairly typical, in terms of low recombination rate regions in the genome.

*Reply: Thanks a lot for the reviewer’s comments. Unfortunately this comment seems to be missing some information.*

12. I do not believe the selection analyses in lines 287 to 330. By definition, a presence variant is going to have a lower pi, because it has a lower population size. The slightly compelling observation from this analysis is that the frequency of the presence variant increases from teosinte to landraces, but this is not uncommon for many variants (due to genetic drift and demographic effects). I didn't understand the significance of the note in lines 311 to 313; is this necessary or can it be explained better? In short, to make this inference properly requires more sophisticated methods than reports of pi and D; simulations might be one approach, but they will require much more careful exposition of null hypotheses.

*Reply: We thank the reviewer for making constructive comments about the selection analysis. We removed the sentence from the previous version of manuscript (lines 311 to 313): “It is worth noting that TDD39103 contained more than 80% missing sites in the flanking region, implying RegionA was possibly localized separately with NOR in Tripsacum.” Because it doesn’t contribute to this paragraph, the phenomenon “RegionA was localized separately with NOR in Tripsacum” was described and discussed in the section of Discussion, so this sentence was a bit redundant here. We argued that RegionA was under selection during domestication and adaptation mainly based on the following evidence: (1) The proportions of RegionA-presence line exhibited a progressively increasing enrichment along with the maize domestication and improvement (from 0.29 to 0.48 to 0.83). (2) There is an excess of negative values of Tajima’s D in RegionA for maize landrace and inbreds (-0.9695, -1.7032, respectively, fig. 5c), suggests an overall excess of lowfrequency mutations. (3) We scanned the signatures of selective sweeps for maize landrace and inbreds, using a site frequency spectra (SFS)-based method by SweeD [16], the composite likelihood ratio (CLR) was calculated with grid sizes of 50kb across each chromosome. We then determined the significance threshold to reject the null model of neutrality based on the demography of maize domestication [17]. More specifically, this demographic model estimates an ancestral effective population size of Na about 123,000, and maize split from teosinte about 15,000 generations ago with an initial size of about 5% of the ancestral Na, maize experienced an exponential population growth after its split from teosinte, reaching a final modern effective population size of 2.98Na. The gene flow from teosinte to maize was estimated to be Mtm = 1.1 x 10-5 x Na migrants per generation, from maize to teosinte was estimated to be Mmt = 1.4 x 10-5 x Na migrants per generation. The mutation rate per year (μ) used here is 3e-8, assuming no recombination. In fact, the effective population size (Ne) in maize is difficult to estimate because of rapid demographic change during and post domestication, here we ran the models with a set of different Ne values (100k, 500k, and 1M). We used Hudson’s ms [18] to perform coalescent simulations and 10,000 1- Mb simulation datasets were generated, the simulation datasets were used as inputs for SweeD. The highest 99.99th percentile of the CLR distribution of simulation datasets was used as a significance threshold for real data (CLR = 4.971903, 5.053264 and 5.127507 for Ne values = 100k, 500k, and 1M). For modern inbreds, which are different from a population at equilibrium, we considered genomic regions with the top 5% of CLR scores as a significance threshold (CLR = 6.258513). Finally, we found the significant signature of selection inside the RegionA for landrace and inbred (Fig. 5d, Supplementary Fig. 10e-g), which rejected the null model of neutrality. Scripts used for simulation were as follows: ms 16 10000 -t 14760 -I 2 5 11 0 -m 1 2 6.888 -m 2 1 5.412 -g 2 86.9681582415 -n 2 0.813 -eg 0.03150406504 2 0.0 -ej 0.03150406504 2 1 -p 10 > 100k_lan.ms ms 16 10000 -t 14760 -I 2 5 11 0 -m 1 2 6.888 -m 2 1 5.412 -g 2 138.054832624 -n 2 4.065 -eg 0.03150406504 2 0.0 -ej 0.03150406504 2 1 -p 10 > 500k_lan.ms ms 16 10000 -t 14760 -I 2 5 11 0 -m 1 2 6.888 -m 2 1 5.412 -g 2 160.056665711 -n 2 8.13 -eg 0.03150406504 2 0.0 -ej 0.03150406504 2 1 -p 10 > 1M_lan.ms Taken together, all the above evidence provided the support that RegionA was targeted by positive selection. We added these details in Methods of revised manuscript and rephrased the selection test part in the main text read as "...Here we scanned the signatures of selective sweeps for maize landrace and inbreds, using a site frequency spectra (SFS)-based method by SweeD [16], the composite likelihood ratio (CLR) was calculated with grid sizes of 50kb across each chromosome. We then determined the significance threshold to reject the null model of neutrality based on the demography of maize domestication [17]....".*

13. Line 344-347. I don't understand the significance of looking at heterochromatic modifiers in this context, since they aren't discussed more carefully. It is not that it is in any way incorrect to report the data, but they don't contribute to the story.

*Reply: In this section, our results confirmed that RegionA genes showed the additive inheritance mode, with respect to both transcriptome and epigenetic modification. Given the epigenetic modifications assayed in our study have been well acknowledged to be positively related to gene expression [19,20], the consistent pattern of modification with the transcription indeed provides additional support to the additive mode of RegionA.*

14. Line 348 - I presumed nonlinear outliers are non-additive (or mid-parent) values? This could be worded more clearly.

*Reply: Thank you for your comments. We’ve changed nonlinear outliers to non-additive for the whole paragraph (Line 356-382).*

15. In my view, lines 353 to 355 doesn't implicate Region A in any particularly way. Is region A particularly prone - relative to the genome pattern - to have such outliers?

*Reply: Thank you for your comments. We re-conducted this part of analysis to address this point. In this version of manuscript, we applied a more stringent criterion for identifying B73 specific PAV genes to Mo17. First, we identified a total of 3,723 B73 singleton genes using the Genetribe pipeline [21]. Then, we filtered those with more than 30% CDS regions covered by Mo17 resequencing reads. Finally, we obtained 911 B73 specific PAV genes, 43 of them are in RegionA. We calculated the gene expression and made comparisons among B73/Mo17 hybrids and B73 for different tissues, only expressed genes (FPKM ≧ 1 in at least one sample) were retained. For tissues consisting of diploid cells, B73 specific genes showing a log2fc > 0 in hybrid than B73 were classified as non-additive (higher) genes, while those showing log2fc < -2 were classified as non-additive (lower) genes. For endosperm (triploid cells), B73 specific genes showing log2fc(BM/B73) > log2(2/3)+1 or log2fc(MB/B73) > log2(1/3)+1 were classified as non-additive (higher) genes, while those showing log2fc(BM/B73) < log2(2/3)-1 or log2fc(MB/B73) < log2(1/3)-1 were classified as non-additive (lower) genes. We identified the non-additive genes for RegionA and compared to the genome pattern, there were significant differences in the proportion of non-additive genes in few tissues compared with the whole genome pattern, but the differences were very mild (Supplementary Table 5). The mild significance may be due to the low number of Region genes shown to be non-additive, and therefore we did not follow up on further characterization of them.*

16. I am concerned about the analysis of NILs. If I understand correctly, the filtering mechanism cannot account for trans-acting effects in imperfect backgrounds. (This is Mo17 alleles in the non-A region of the B_m line that drive observations of differential expression.) As such, I think concluding that the A region plays a role in the 486 DEGs is possibly exaggerated and perhaps wrong.

*Reply: We acknowledge that the background effects of our NILs were not perfectly eliminated, since the repressed recombination around NOR makes the background difficult to be purified even after several generations. For now we don’t have more perfect experimental materials in hand to re-conduct analysis. Alternatively, we assume that the majority of these negative positive DEGs could have been filtered out by these following steps. Firstly, we only retained the overlapping up- or down-regulated DEGs between BM and MB datasets, it mutually eliminated the impact of their respective imperfect backgrounds. Secondly, to reduce the impact of trans-acting effects as much as possible, we extracted the eQTLs locus (a 1-kb window centered on top SNPs in the eQTLs) of B73/Mo17 RIL population [22] that overlapped with snps between our B73/Mo17 NILs. Totally 946 target genes of overlapped eQTLs locus were defined as false positive genes in the imperfect backgrounds. After filtering these genes out from 486 DEGs, finally we got 473 DEGs and did downstream analysis (Supplementary Fig. 15d). This filtering also indicates the earlier DEGs overlaps between BM and MB datasets performed well to reduce the background effect. After the additional eQTL filtering, we believe the final dataset of 473 DEGs conservatively represents the PAV effect on the maize transcriptome.*

17. Line 402. One of the things about PAVs - and their apparent rarity - is not only one of semantics (see point #1 above) but the lack of good data. I wonder if this point, which is made nicely in lines 430 to 432, could be moved here?

*Reply: Thanks for your constructive suggestion. We agree that the previous lack of long reads sequencing is one of the reasons for the rarity of large size PAVs. We have revised the text accordingly to address this point.*

18. I like the equanimity of lines 456 to 460. Indeed, while I agree with both models, I find the evidence for introgression to be tentative at best.

*Reply: Thanks for your comments.*

Reviewer #2:

In this manuscript, Huang et al. updated the maize B73 reference genome using Hi-C data and conducted genomic analyses on big SVs. The authors found some interesting SV patterns in domestication of maize and zea. I think the manuscript was well written. However, many parts of the evolutionary genetic analyses were unprofessional and conclusions draw form it was misleading and incorrect. I also have other major and minor comments for the authors to consider.

Major: 1. The authors seem don't familiar with the literature on structural variation and on zea evolution. Some fundamental papers on SV and plant evolution and on zea evolution were not referred in this paper, so that the authors used "novel", "unknown" and similar words across the paper. I recommend the authors to rewrite the manuscript, especially the introduction and discussion part, based on a better understanding on the recent process in the field.

*Reply: Thank the reviewer for the constructive comments and suggestions. More references have been included in the revised manuscript, specific references are listed as follows: [1,2,4,7,23]. We have carefully scrutinized the manuscript, and made corresponding revisions. All the major rewritten has been labeled as red text.*

2. The evolutionary genomic analyses were unprofessional, and the conclusions draw form it was misleading and incorrect at many places. For example, the authors calculated the population mutation rate and genetic diversity at the big SV region and argued that the SV regions were low in diversity and under selection. A given SV is a single locus (like a SNP) with all sites highly linked within it. So, it has nothing to do with selection, it is just a common feature of SV. Another example the zea migration part. I don't think the authors had enough samples or analyses to reach this conclusion.

*Reply: Thanks for your suggestions, which is valuable for improving the accuracy of the manuscript. We agree with the reviewer that SV regions are often accompanied by the characteristics of low diversity and high level of linkage disequilibrium. In fact, from lines 312-336, we just reported the population mutation rate and genetic diversity, then made comparison with the flanking region and investigated the phylogenetic relationships, this part was not used as evidence to judge whether RegionA was under selection. As we also responded to the Reviewer 1, we argued that RegionA was under selection during domestication and adaptation mainly based on the following evidence: (1) The proportions of RegionA-presence line exhibited a progressively increasing enrichment along with the maize domestication and improvement (from 0.29 to 0.48 to 0.83). (2) We detected an excess of negative values of Tajima’s D in RegionA for maize landrace and inbreds (-0.9695, -1.7032, respectively, fig. 5c), suggests an overall excess of low-frequency mutations. (3) We scanned the signatures of selective sweeps for maize landrace and inbreds, using a site frequency spectra (SFS)-based method by SweeD [16], the composite likelihood ratio (CLR) was calculated with grid sizes of 50kb across each chromosome. We then determined the significance threshold to reject the null model of neutrality based on the demography of maize domestication [17]. More specifically, this demographic model estimates an ancestral effective population size of Na about 123,000, and maize split from teosinte about 15,000 generations ago with an initial size of about 5% of the ancestral Na, maize experienced an exponential population growth after its split from teosinte, reaching a final modern effective population size of 2.98Na. The gene flow from teosinte to maize was estimated to be Mtm = 1.1 x 10-5 x Na migrants per generation, from maize to teosinte was estimated to be Mmt = 1.4 x 10-5 x Na migrants per generation. The mutation rate per year (μ) used here is 3e-8, assuming no recombination. In fact, the effective population size (Ne) in maize is difficult to estimate because of rapid demographic change during and post domestication, here we ran the models with a set of different Ne values (100k, 500k, and 1M). We used Hudson’s ms [18] to perform coalescent simulations and generated 10,000 1-Mb simulation datasets; these simulation datasets were used as inputs for SweeD to calculate CLR. The highest 99.99th percentile of the CLR distribution of simulation datasets was used as a significance threshold for real data (CLR = 4.971903, 5.053264 and 5.127507 for Ne values = 100k, 500k, and 1M). For modern inbreds, which is different from a population at equilibrium, we considered genomic regions with the top 5% of CLR scores as a significance threshold (CLR = 6.258513). Finally, we found the significant signature of selection inside the RegionA for landrace and inbred (Fig. 5d, Supplementary Fig. 10eg), which rejected the null model of neutrality. Combined all aboved evidence, so that we made the conclusion that RegionA was under selection. We added these details in Methods of revised manuscript and rephrased the selection test part in the main text. For the part of Zea migration, we agree that in addition to the Treemix results, further analysis should be deployed to clarify the gene migration from Tripsacum to maize. According to Reviewer 1’s suggestion, we calculated absolute sequence divergence (dxy) between Tripsacum and Zea in RegionA and compared it to the whole genome. The dxy value of 60.5%~72.2% RegionA windows were below the cutoff of whole genome bottom 5% (Supplementary Figure 9). Since the reduced dxy has been generally considered as the sign of recent gene flow distinguished from shared ancestral variation [14,15], we attribute the dramatically reduced dxy between Tripsacum and Zea at RegionA to the recent coalescence at this region led by introgression. Objectively speaking, combined with our available data, it is difficult to distinguish between introgression and shared ancestral variation. We rephrased several sentences of this part in the main text and carefully discussed these two possible scenarios.*

*Supplementary Figure 9. Absolute sequence divergence (dxy) between Tripsacum and Zea. (a) dxy values between Tripsacum and Zea across maize chromosome 6. RegionA is indicated by two vertical lines. (b) Density plot of dxy value of RegionA windows. The dashed vertical lines indicated the cutoff of the whole genome bottom 5% dxy value.*

Minor: 1. L19: What is "environmental plasticity"?

*Reply: We’ve changed "environmental plasticity" to "environmental adaptation" (line 19).*

2. L27: What does "evolution erosion" mean? Please write clearly using filed-specific words.

*Reply: We’ve rephrased this sentence (Line 25-27): “This PAV is embedded within the nucleolus organizer region (NOR), where the suppressed recombination was found to maintain the PAV against the evolutionary variation”.*

3. L29: Introgression from distant-related species is a very common feature in plants, what is "unknown"?

*Reply: Yes, it is well-known that introgression from distant-related species is not uncommon in plants. Tripsacum is the closest relative outside the Zea genus, but there was no previous report about the contribution from Tripsacum to the speciation of Zea species.*

4. L36: The "fossil" SV?

*Reply: This is a metaphor. RegionA is like a fossil that is embedded within the nucleolus organizer region (NOR), so that it preserves the sequence integrity and provides the opportunity to trace the footprint that may be preserved against the evolution dynamic.*

5. L41: Paper 1,2 have nothing to do with SVs.

*Reply: We have updated reference reads as follows: [24–27].*

6. L42-43: This is incorrect. Please read recent review papers on plant SVs carefully.

*Reply: We have rewritten this sentence as follows: Structural variation mainly includes presence-absence variants (PAV), copy number variants (CNV), inversions, translocations and more complex rearrangement [28,29]. One extreme form of CNV is the presence-absence variation (PAV), in which a particular sequence is present in some individuals while absent in others.*

7. L53-54: This is incorrect too. Please read recent studies in rice, maize, tomato and grape.

*Reply: Thanks for the reviewer’s comments. We have made the change as follows: Nevertheless, a growing number of eukaryotes, such as fungi, algae and plant species, have been reported with large-size PAVs containing functional genes [1,2,30–33].*

8. L55-62: The citations are missing in this paragraph and many of the arguments are wrong.

*Reply: We have revised the text to address your concerns and have updated the references.*

9. L63-781: The whole introduction is on PAV not SV, but the paper was titled with SV. Please note that PAV ≠SV.

*Reply: We have changed the title with "Megabase-scale presence-absence variation with Tripsacum origin was under selection during maize domestication and adaptation".*

10. L79 and following in the same paragraph: Please cite the original papers in each sentence.

*Reply: Thanks for your comments. We agree and have updated the citation.*

11. L115: This had been done several times already in previous publications, why again?

*Reply: Thanks a lot for the reviewer’s comments. Although the identification of PAVs between B73 and Mo17 had been done in previous publications, those reports were based on previous versions of the B73 reference genome, which unfortunately contains low quality scaffolding and assembly errors in this region. In our study, we updated the assembly of B73 chromosome 6, so that we characterized not only the accurate length of this PAV, but more importantly unearthed the novel finding about its location embedded within the NOR region.*

12. L116-182: Why region A-E were chosen, there was very strong bias that comparing Mo17 and B73 and used the PAV markers in the multi-species diversity panel.

*Reply: Thanks for your comments. B73 and Mo17 are the most common pair of maize inbred lines in many genetic and molecular studies, they represent two best-known maize variety groups (Reid yellow dent and Lancaster). Moreover, their high-quality reference genome (using single-molecule sequencing and BioNano optical-mapping technologies) are both available which improve the accuracy of our identification. Our study only focuses on these largest PAVs between B73 and Mo17, and shows their diversity in the natural diversity panel, but they do not represent the general profile of large PAVs in the maize diversity population.*

13. L182-L254: The analyses done here was not enough to support the authors arguments.

*Reply: Thanks for your comments. As Reviewer1 also raised a similar concern and also a constructive suggestion about this point, we calculated absolute sequence divergence (dxy) between Tripsacum and Zea in RegionA and compared it to the whole genome, as the Reviewer 1 suggested. The result shows a highly genetic similarity between Tripsacum and Zea at RegionA, and this result can be served as another evidence for introgression. We also carefully discussed the two plausible scenarios of introgression and shared ancestral variation, please see lines 474-499.*

14. L286-330: Please see my major comment 2.

*Reply: Thanks a lot for the reviewer’s comment. To address this point, we have revised the part of the selection test, which made a more careful exposition of null hypotheses for coalescent simulations, please see major reply 2 for more details.*

15. L331-355: This is a common feature of PAV.

*Reply: Large-size PAVs (megabase-scale) have been occasionally described in plants, but the number of genes within them is usually limited. It is unusual that RegionA is a gene-rich region that contains 70 protein-coding genes (21.5 genes per megabase compared to a genome-wide average of 18.7 genes per megabase). Although there are some reports focusing on the expression mode of PAV genes at the genome-wide level, the specific focus on such a large region containing 70 continuous PAV genes has been rarely explored. So we think it is intriguing and worth more follow up study on it.*

16. L356: What is "environmental fitness", do the authors mean environmental adaptation?

*Reply: We’ve changed "environmental fitness" to "environmental adaptation".*

17. L433: See my major comment 2.

*Reply: Please see our detailed response to the major comment 2.*

18. L526: There is a non-gap assembly of B73 based on deep Pacbio sequencing and scaffolding (V5). Why the authors chose to update an old version?

*Reply: Thanks for your suggestions. We acknowledge that the v5 genome has been released recently, but our research had been conducted just before the v5 was released, hence only the v4 reference genome was available at that time. However, we believe this does not affect the results and conclusions drawn from our study.*

19. Fig. 6 is misleading!

*Reply: Thanks a lot for the reviewer’s comments. More details were added to the figure legend to clarify these two possible scenarios of the evolution of the RegionA, which are also agreed by Reviewer 1. Future research is required to identify either of the scenarios to be the correct evolutionary path of the RegionA, though it may be out of the scope of this study.*

*Reference*

*1. Zhou Y, Minio A, Massonnet M, Solares E, Lv Y, Beridze T, et al. The population genetics of structural variants in grapevine domestication. Nat Plants. 2019;5:965–79. http://dx.doi.org/10.1038/s41477-019-0507-8.*

*2. Kou Y, Liao Y, Toivainen T, Lv Y, Tian X, Emerson JJ, et al. Evolutionary Genomics of Structural Variation in Asian Rice (Oryza sativa) Domestication. Mol Biol Evol. 2020;37:3507–24.* [*http://dx.doi.org/10.1093/molbev/msaa185*](http://dx.doi.org/10.1093/molbev/msaa185)*.*

*3. Han J-J, Jackson D, Martienssen R. Pod corn is caused by rearrangement at the Tunicate1 locus. Plant Cell. 2012;24:2733–44.* [*http://dx.doi.org/10.1105/tpc.112.100537*](http://dx.doi.org/10.1105/tpc.112.100537)*.*

*4. Crow T, Ta J, Nojoomi S, Aguilar-Rangel MR, Torres Rodríguez JV, Gates D, et al. Gene regulatory effects of a large chromosomal inversion in highland maize. PLoS Genet. 2020;16:e1009213. http://dx.doi.org/10.1371/journal.pgen.1009213.*

*5. Wang L, Beissinger TM, Lorant A, Ross-Ibarra C, Ross-Ibarra J, Hufford MB. The interplay of demography and selection during maize domestication and expansion. Genome Biol. 2017;18:215.* [*http://dx.doi.org/10.1186/s13059-017-1346-4*](http://dx.doi.org/10.1186/s13059-017-1346-4)*.*

*6. Bilinski P, Albert PS, Berg JJ, Birchler JA, Grote MN, Lorant A, et al. Parallel altitudinal clines reveal trends in adaptive evolution of genome size in Zea mays. PLoS Genet. 2018;14:e1007162. http://dx.doi.org/10.1371/journal.pgen.1007162.*

*7. Roessler K, Muyle A, Diez CM, Gaut GRJ, Bousios A, Stitzer MC, et al. The genome-wide dynamics of purging during selfing in maize. Nat Plants. 2019;5:980–90.* [*http://dx.doi.org/10.1038/s41477-019-0508-7*](http://dx.doi.org/10.1038/s41477-019-0508-7)*.*

*8. Anderson SN, Stitzer MC, Brohammer AB, Zhou P, Noshay JM, O’Connor CH, et al. Transposable elements contribute to dynamic genome content in maize. Plant J. 2019;100:1052–65. http://dx.doi.org/10.1111/tpj.14489.*

*9. Sun S, Zhou Y, Chen J, Shi J, Zhao H, Zhao H, et al. Extensive intraspecific gene order and gene structural variations between Mo17 and other maize genomes. Nature Genetics. 2018. p. 1289–95. http://dx.doi.org/10.1038/s41588-018-0182-0*

*10. Xie C, Tammi MT. CNV-seq, a new method to detect copy number variation using high-throughput sequencing. BMC Bioinformatics. 2009;10:80. http://dx.doi.org/10.1186/1471-2105-10-80*

*11. Hufford MB, Seetharam AS, Woodhouse MR. De novo assembly, annotation, and comparative analysis of 26 diverse maize genomes. bioRxiv. biorxiv.org; 2021; https://www.biorxiv.org/content/10.1101/2021.01.14.426684v1.abstract*

*12. Wang K, Yang Z, Shu C, Hu J, Lin Q, Zhang W, et al. Higher axial-resolution and sensitivity pachytene fluorescence in situ hybridization protocol in tetraploid cotton. Chromosome Res. 2009;17:1041–50. http://dx.doi.org/10.1007/s10577-009-9085-3*

*13. Martin SH, Davey JW, Jiggins CD. Evaluating the Use of ABBA–BABA Statistics to Locate Introgressed Loci. Mol Biol Evol. Oxford Academic; 2014;32:244–57. https://academic.oup.com/mbe/article-abstract/32/1/244/2925550*

*14. Smith J, Kronforst MR. Do Heliconius butterfly species exchange mimicry alleles? Biol Lett. 2013;9:20130503. http://dx.doi.org/10.1098/rsbl.2013.0503*

*15. Martin SH, Davey JW, Jiggins CD. Evaluating the use of ABBA-BABA statistics to locate introgressed loci. Mol Biol Evol. 2015;32:244–57. http://dx.doi.org/10.1093/molbev/msu269*

*16. Pavlidis P, Živkovic D, Stamatakis A, Alachiotis N. SweeD: likelihood-based detection of selective sweeps in thousands of genomes. Mol Biol Evol. 2013;30:2224–34. http://dx.doi.org/10.1093/molbev/mst112*

*17. Beissinger TM, Wang L, Crosby K, Durvasula A, Hufford MB, Ross-Ibarra J. Recent demography drives changes in linked selection across the maize genome. Nature Plants. 2016;2:16084. https://doi.org/10.1038/nplants.2016.84*

*18. Hudson RR. Generating samples under a Wright–Fisher neutral model of genetic variation. Bioinformatics. Oxford Academic; 2002;18:337–8. https://academic.oup.com/bioinformatics/article/18/2/337/225783*

*19. He G, Chen B, Wang X, Li X, Li J, He H, et al. Conservation and divergence of transcriptomic and epigenomic variation in maize hybrids. Genome Biol. 2013;14:R57. http://dx.doi.org/10.1186/gb-2013-14-6-r57.*

*20. Dong X, Zhang M, Chen J, Peng L, Zhang N, Wang X, et al. Dynamic and Antagonistic Allele-Specific Epigenetic Modifications Controlling the Expression of Imprinted Genes in Maize Endosperm. Mol Plant. 2017;10:442–55. http://dx.doi.org/10.1016/j.molp.2016.10.007*

*21. Chen Y, Song W, Xie X, Wang Z, Guan P, Peng H, et al. A CollinearityIncorporating Homology Inference Strategy for Connecting Emerging Assemblies in the Triticeae Tribe as a Pilot Practice in the Plant Pangenomic Era. Mol Plant. 2020;13:1694–708. http://dx.doi.org/10.1016/j.molp.2020.09.019.*

*22. Li L, Petsch K, Shimizu R, Liu S, Xu WW, Ying K, et al. Mendelian and nonMendelian regulation of gene expression in maize. PLoS Genet. 2013;9:e1003202. http://dx.doi.org/10.1371/journal.pgen.1003202.*

*23. Fang Z, Pyhäjärvi T, Weber AL, Dawe RK, Glaubitz JC, González J de JS, et al. Megabase-scale inversion polymorphism in the wild ancestor of maize. Genetics. 2012;191:883–94. http://dx.doi.org/10.1534/genetics.112.138578.*

*24. Messing J, Dooner HK. Organization and variability of the maize genome. Curr Opin Plant Biol. 2006;9:157–63. http://dx.doi.org/10.1016/j.pbi.2006.01.009.*

*25. Buckler ES, Gaut BS, McMullen MD. Molecular and functional diversity of maize. Curr Opin Plant Biol. 2006;9:172–6. http://dx.doi.org/10.1016/j.pbi.2006.01.013.*

*26. Sun S, Zhou Y, Chen J, Shi J, Zhao H, Zhao H, et al. Extensive intraspecific gene order and gene structural variations between Mo17 and other maize genomes. Nat Genet. 2018;50:1289–95. http://dx.doi.org/10.1038/s41588-018-0182-0.*

*27. Wellenreuther M, Mérot C, Berdan E, Bernatchez L. Going beyond SNPs: The role of structural genomic variants in adaptive evolution and species diversification. Mol Ecol. 2019;28:1203–9. http://dx.doi.org/10.1111/mec.15066.*

*28. Alkan C, Coe BP, Eichler EE. Genome structural variation discovery and genotyping. Nature Reviews Genetics. 2011. p. 363–76. http://dx.doi.org/10.1038/nrg2958.*

*29. Lei L, Goltsman E, Goodstein D, Wu GA, Rokhsar DS, Vogel JP. Plant PanGenomics Comes of Age. Annu Rev Plant Biol. 2021;* [*http://dx.doi.org/10.1146/annurev-arplant-080720-105454*](http://dx.doi.org/10.1146/annurev-arplant-080720-105454)*.*

*30. Marroni F, Pinosio S, Morgante M. Structural variation and genome complexity: is dispensable really dispensable? Curr Opin Plant Biol. 2014;18:31–6.* [*http://dx.doi.org/10.1016/j.pbi.2014.01.003*](http://dx.doi.org/10.1016/j.pbi.2014.01.003)*.*

*31. Read BA, Kegel J, Klute MJ, Kuo A, Lefebvre SC, Maumus F, et al. Pan genome of the phytoplankton Emiliania underpins its global distribution. Nature. 2013;499:209–13. http://dx.doi.org/10.1038/nature12221.*

*32. Yue J-X, Li J, Aigrain L, Hallin J, Persson K, Oliver K, et al. Contrasting evolutionary genome dynamics between domesticated and wild yeasts. Nat Genet. 2017;49:913–24. http://dx.doi.org/10.1038/ng.3847.*

*33. Alonge M, Wang X, Benoit M, Soyk S, Pereira L, Zhang L, et al. Major Impacts of Widespread Structural Variation on Gene Expression and Crop Improvement in Tomato. Cell. 2020;182:145–61.e23. http://dx.doi.org/10.1016/j.cell.2020.05.021.*

**Second round of review**

**Reviewer 1**

Thank you to the authors for thorough revisions. I continue to think that the authors have found an interesting region that merits attention and deserves publication. I provide some minor comments below! I try to be clear as to what seems necessary vs. entirely optional.

L. 72 - fractionation is usually a result of polyploidy, not introgression. I'd remove fractionation here, but it's obviously not a major point that the authors should feel to disagree with.

L. 78 - Again, it would be fair to say that few large PAVs have been evaluated, but potentially misleading to say large PAVs are rare because their isn't much information on them thus far. (And inbreds like B73 and Mo17 probably have very few relative to real diploids with larger genome sizes…) Can you reword slightly to reflect this important difference?

L. 111 - I very much like the discussion paragraph that points out that RegionA could be explained by introgression or lineage sorting. I think the favored interpretation of introgression is fair, but I'm not sure I'd call it "strong evidence". Can this wording be softened?

L. 113 - "demonstrated" that it was a product of selection is pretty strong. I'll say more about this below, but how about softer language like "may be consistent" with selection?

L. 123 "that was previously mentioned". I don't think it was. Might want to clarify. Sorry if I missed it!

L 186. "Et cetera" isn't appropriate. Either list more accessions or say "and others" or something like that.

L. 232 and following. I think that Figure S7 has the information on this, but what I really want to know is whether Region A has a significant enrichment of Tse transposons vs. the rest of the genome. I think a simple t-test (or similar) based on the histograms in S7 would be nice to report somewhere here!

L 268 - just an fyi: a couple of citations here weren't formatted by the reference software.

L 273 - I'm a bit worried by the dxy and selective sweep analyses. Hopefully my worry is misplaced, but I wasn't 100% sure what was done. Here's the problem: if you do dxy and selective sweep mapping on a sample that both includes and does not include RegionA, then there will be a strong bias to find sweeps in region A and to find low dxy. The reason for this is just a sample size phenomenon - there are fewer samples with a more recent history for RegionA than for the rest of the genome. That said, I'm not sure what else you can do … other than some very complicated popgen approaches … because there isn't an "off the shelf" way to deal with this problem. So, I think what you have done is reasonable. My suggestion and solution is to be transparent about this issue, to be clear about what samples were tested (just samples with Region A or a mixed sample?), to admit potential biases (depending on the samples) and to soften language about both dxy and sweep. I gave an example of softened language above for L. 113 above. I'll try to point out other areas that can be modified a bit, too, with the intent to try to be helpful.

L 327 - I'm not really sure what this sentence means. Is it necessary?

L. 337 and following - I do not advocate large changes in analyses, but please make it clear if sweep analyses were done on a mixed population (that contained both RegionA and did not) or only on a sample with RegionA. If the former (mixed) how were the RegionA regions dealt with to be comparable with other non-PAV windows? Somewhere in here, it might be worth admitting there is a potential bias, too, as mentioned above.

L. 363 - Sorry if I missed this, and I don't think this needs to be included. But I found myself wondering if the level of expression (averaged across tissues, I guess?) is similar for RegionA genes vs. the genome-wide average. The reason I ask is that translocated genes in maize are often lowly expressed… Again, I'm mostly curious, and I don't think this needs to be included if it's difficult to do.

L. 475 - Again, important to be clear that this drastic reduction could be due to biases in the way the analysis was done. (With apologies if this was not done a mixed PAV sample).

- Given the really strong evidence that another SV (the maize inversion, but not a PAV) is associated with plant growth, highland adaptation, flowering time, introgression and other phenomena, I continue to be a bit surprised that it isn't mentioned more prominently in the Discussion as a fitting parallel and context for the findings in this paper. It's ultimately the authors' decision, of course (and therefore entirely optional!, but the inversion is widely known and putting in this context would add value to the paper, in my opinion.

To sum: I think the phenomenon of the RegionA PAV is really neat, there is extensive and interesting data (FISH, gene expression, etc.), and it deserves to be published, in my view.

**Reviewer 2**

I am pleased to see the revisions the authors made on this manuscript. In general, I think the manuscript is almost ready for publication. However, I also have some minor comments for the authors to consider.

Introduction:

The introduction part still needs more work. Please read classical and review papers in maize (10.1126/science.1107891) and structural variants (for example 10.1038/ng2042 and 10.1038/s41477-018-0210-1) and rewrite it.

Figure legends of figure2 are unclear, please check it.

**Authors Response**

**Point-by-point responses to the reviewers’ comments:**

Reviewer #1:

Thank you to the authors for thorough revisions. I continue to think that the authors have found an interesting region that merits attention and deserves publication. I provide some minor comments below! I try to be clear as to what seems necessary vs. entirely optional.

1. L. 72 - fractionation is usually a result of polyploidy, not introgression. I'd remove fractionation here, but it's obviously not a major point that the authors should feel to disagree with.

*Reply: We removed “fractionation” here.*

2. L. 78 - Again, it would be fair to say that few large PAVs have been evaluated, but potentially misleading to say large PAVs are rare because their isn't much information on them thus far. (And inbreds like B73 and Mo17 probably have very few relative to real diploids with larger genome sizes…) Can you reword slightly to reflect this important difference?

*Reply: We have rewritten this sentence as follows:*

*“However, due to the current lack of evaluation of large size PAVs in plants and their own rarity, it is still ambiguous if interspecific transfer or genome fractionation directly links to the large size PAVs.”*

3. L. 111 - I very much like the discussion paragraph that points out that RegionA could be explained by introgression or lineage sorting. I think the favored interpretation of introgression is fair, but I'm not sure I'd call it "strong evidence". Can this wording be softened?

*Reply: We have changed “Strong evidence” to “A series evidence”*

4. L. 113 - "demonstrated" that it was a product of selection is pretty strong. I'll say more about this below, but how about softer language like "may be consistent" with selection?

*Reply: We have rewritten this sentence as follows:*

*“Lastly, we found this Tripascum-originated PAV may be consistent with selection during maize domestication and improvement”.*

5. L. 123 "that was previously mentioned". I don't think it was. Might want to clarify. Sorry if I missed it!

*Reply: The sliding-window method we used here was described in the previous publication (Sun et al. 2018).*

6. L 186. "Et cetera" isn't appropriate. Either list more accessions or say "and others" or something like that.

*Reply: We have rewritten this sentence as follows:*

*“B73 RegionA showed well collinearity with the genome of PH207, B104, and 19 out of 24 other NAM founders besides B73 (Additional file 1: Figure S4), but no collinearity with Mo17, W22 and remaining NAM founders, consistent with the presence-bin proportion pattern shown in Fig. 1d.”.*

7. L. 232 and following. I think that Figure S7 has the information on this, but what I really want to know is whether Region A has a significant enrichment of Tse transposons vs. the rest of the genome. I think a simple t-test (or similar) based on the histograms in S7 would be nice to report somewhere here!

*Reply: We replaced the histograms with the boxplot as follows, RegionA showed a significantly lower hits number than the other random region (Mann–Whitney U test, MWU, only significant comparisons were shown).*

*(f) Comparisons of genome wide hits number for LTR-RTs in RegionA and 5 random region. RegionA LTR-RTs showed significantly lower hits number compared with other random region (Mann–Whitney U test, MWU, only significant comparisons were shown).*

8. L 268 - just an fyi: a couple of citations here weren't formatted by the reference software.

*Reply: The references have been reformatted, thanks.*

9. L 273 - I'm a bit worried by the dxy and selective sweep analyses. Hopefully my worry is misplaced, but I wasn't 100% sure what was done. Here's the problem: if you do dxy and selective sweep mapping on a sample that both includes and does not include RegionA, then there will be a strong bias to find sweeps in region A and to find low dxy. The reason for this is just a sample size phenomenon - there are fewer samples with a more recent history for RegionA than for the rest of the genome. That said, I'm not sure what else you can do … other than some very complicated popgen approaches … because there isn't an "off the shelf" way to deal with this problem. So, I think what you have done is reasonable. My suggestion and solution is to be transparent about this issue, to be clear about what samples were tested (just samples with Region A or a mixed sample?), to admit potential biases (depending on the samples) and to soften language about both dxy and sweep. I gave an example of softened language above for L. 113 above. I'll try to point out other areas that can be modified a bit, too, with the intent to try to be helpful.

*Reply: We totally understand the reviewer’s concern. In fact, all the selection and dxy test were performed only using RegionA-presence samples to avoid bias. We have added these details in the revised manuscript.*

10. L 327 - I'm not really sure what this sentence means. Is it necessary?

*Reply: We removed this sentence from the revised manuscript.*

11. L. 337 and following - I do not advocate large changes in analyses, but please make it clear if sweep analyses were done on a mixed population (that contained both RegionA and did not) or only on a sample with RegionA. If the former (mixed) how were the RegionA regions dealt with to be comparable with other non-PAV windows? Somewhere in here, it might be worth admitting there is a potential bias, too, as mentioned above.

*Reply: Thank you for your comments. The sweep analyses were done on the RegionA-presence samples. We have added these details to clarify this point in the revised manuscript.*

12. L. 363 - Sorry if I missed this, and I don't think this needs to be included. But I found myself wondering if the level of expression (averaged across tissues, I guess?) is similar for RegionA genes vs. the genome-wide average. The reason I ask is that translocated genes in maize are often lowly expressed… Again, I'm mostly curious, and I don't think this needs to be included if it's difficult to do.

*Reply: To address this point, we performed comparison for expression level between RegionA genes and genome-wide in 23 tissues using a previously published dataset (Walley et al. 2016). As you speculated, the RegionA genes indeed showed significantly lower expression levels than the rest of genome-wide genes in all tissues we tested (Two-sided Wilcoxon ranked sum tests).*

*13. Comment:*

*L. 475 - Again, important to be clear that this drastic reduction could be due to biases in the way the analysis was done. (With apologies if this was not done a mixed PAV sample)*

*13. Reply:*

*Thank you for your comments. The dxy analyses were done on the RegionA-presence samples. We have added these details accordingly in the revised manuscript.*

14. Given the really strong evidence that another SV (the maize inversion, but not a PAV) is associated with plant growth, highland adaptation, flowering time, introgression and other phenomena, I continue to be a bit surprised that it isn't mentioned more prominently in the Discussion as a fitting parallel and context for the findings in this paper. It's ultimately the authors' decision, of course (and therefore entirely optional!, but the inversion is widely known and putting in this context would add value to the paper, in my opinion.

*Reply: Thanks for your great suggestion. We added these contexts to the Discussion.*

*15. To sum: I think the phenomenon of the RegionA PAV is really neat, there is extensive and interesting data (FISH, gene expression, etc.), and it deserves to be published, in my view.*

*Reply: Thank you!*

Reviewer #2:

I am pleased to see the revisions the authors made on this manuscript. In general, I think the manuscript is almost ready for publication. However, I also have some minor comments for the authors to consider.

1. Introduction: The introduction part still needs more work. Please read classical and review papers in maize (10.1126/science.1107891) and structural variants (for example 10.1038/ng2042 and 10.1038/s41477-018-0210-1) and rewrite it.

*Reply: We have done further polish on the introduction, more references, including the ones the Reviewer suggested, have been added.*

2. Figure legends of figure2 are unclear, please check it.

*Reply: Figure 2 legend has been revised.*

*Sun, Silong, Yingsi Zhou, Jian Chen, Junpeng Shi, Haiming Zhao, Hainan Zhao, Weibin Song, et al. 2018. “Extensive Intraspecific Gene Order and Gene Structural Variations between Mo17 and Other Maize Genomes.” Nature Genetics. https://doi.org/10.1038/s41588-018-0182-0.*

*Walley, Justin W., Ryan C. Sartor, Zhouxin Shen, Robert J. Schmitz, Kevin J. Wu, Mark A. Urich, Joseph R. Nery, et al. 2016. “Integration of Omic Networks in a Developmental Atlas of Maize.” Science 353 (6301): 814–18. https://doi.org/10.1126/science.aag1125.*
